# Please do not recycle! Translation reinitiation in microbes and higher eukaryotes

**DOI:** 10.1093/femsre/fux059

**Published:** 2017-12-21

**Authors:** Stanislava Gunišová, Vladislava Hronová, Mahabub Pasha Mohammad, Alan G Hinnebusch, Leoš Shivaya Valášek

**Affiliations:** 1 Laboratory of Regulation of Gene Expression, Institute of Microbiology ASCR, Videnska 1083, Prague, 142 20, the Czech Republic; 2 Laboratory of Gene Regulation and Development, Eunice Kennedy Shriver National Institute of Child Health and Human Development, NIH, Bethesda, MD 20892, USA

**Keywords:** translation reinitiation, termination-reinitiation, uORF, GCN4, eIF2, eIF3

## Abstract

Protein production must be strictly controlled at its beginning and end to synthesize a polypeptide that faithfully copies genetic information carried in the encoding mRNA. In contrast to viruses and prokaryotes, the majority of mRNAs in eukaryotes contain only one coding sequence, resulting in production of a single protein. There are, however, many exceptional mRNAs that either carry short open reading frames upstream of the main coding sequence (uORFs) or even contain multiple long ORFs. A wide variety of mechanisms have evolved in microbes and higher eukaryotes to prevent recycling of some or all translational components upon termination of the first translated ORF in such mRNAs and thereby enable subsequent translation of the next uORF or downstream coding sequence. These specialized reinitiation mechanisms are often regulated to couple translation of the downstream ORF to various stimuli. Here we review all known instances of both short uORF-mediated and long ORF-mediated reinitiation and present our current understanding of the underlying molecular mechanisms of these intriguing modes of translational control.

## Teaser

This review covers one of many means of gene-specific translational control called translational reinitiation that (i) occurs on mRNAs carrying more than one ORF, (ii) can be mediated by either post-termination 40S or 70S/80S ribosomes, and (iii) is highly regulated in response to various stresses and many other intra- or extracellular signals.

## INTRODUCTION

mRNA translation is a cyclic process regularly alternating four basic phases: initiation, elongation, termination and ribosomal recycling. It is also one of the most energy-consuming processes in the cells. Therefore, to minimize energy expenditure, each cycle of protein synthesis reuses the components of the translation machinery that have been already used in the previous cycle, including mRNAs. The initiation phase is the most intricate process of all and its coordination relies on several initiation factors (IFs in bacteria and eIFs in eukaryotes). In fact, this is also the phase where prokaryotes and eukaryotes differ the most. The major differences lie in (i) the number of initiation factors that they utilize (3 IFs in bacteria compared to at least 12 eIFs in eukaryotes) and (ii) the way the pre-initiation complexes (PICs) assemble and recognize the AUG initiation codon. In prokaryotes, the formyl-methionyl-tRNA (fMet-tRNA_f_^Met^) can associate with the 30S subunit on its own and AUG is placed directly into the ribosomal P-site via base pairing of the Shine-Dalgarno (SD) sequence in the mRNA, located upstream of the AUG, with the very 3^΄^ end of 16S rRNA. In eukaryotes, the Met-tRNA_i_^Met^ does not associate with the 40S subunit on its own, mRNAs carry the 5^΄^ 7-methylguanosine cap that is recognized by specific initiation factors needed for mRNA recruitment by the PIC, the mRNA 5^΄^ leader sequences are generally longer and often contain stable secondary structures, and AUG is recognized by ribosomal scanning in the 5^΄^ to 3^΄^ direction with the help of RNA helicases.

**Figure 1.**
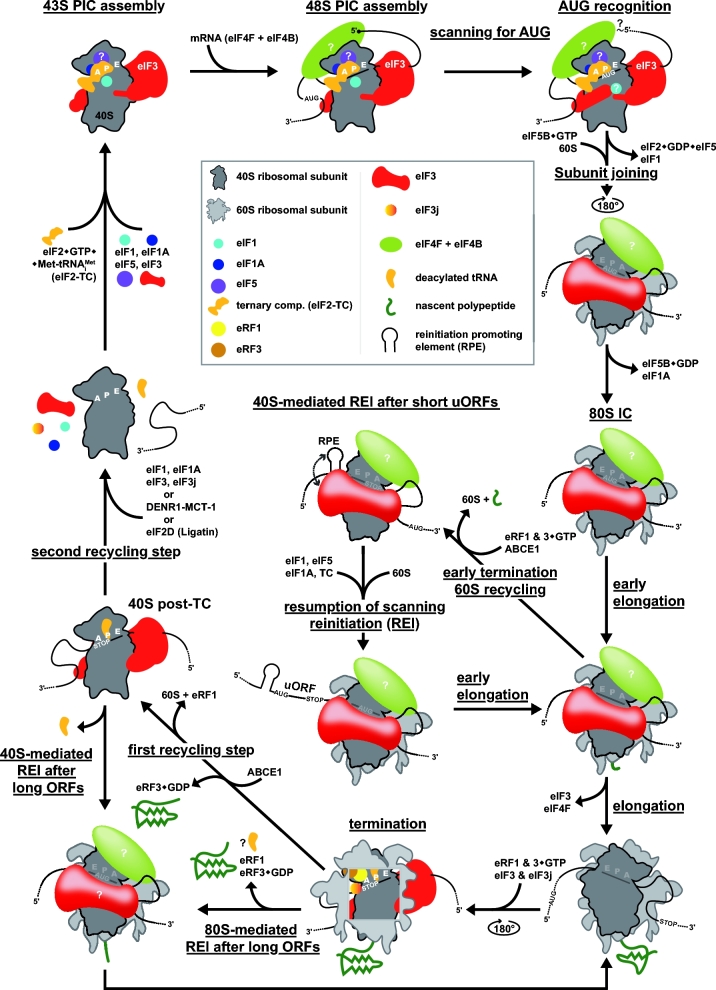
Model of the entire translational cycle with two basic ways of translation reinitiation: (i) the 40S-mediated REI after short versus long uORFs and (ii) the 80S-mediated REI after long ORFs. For details, see the main text (adapted from Valasek *et al.* 2017).

In this review, we focus in more detail on the initiation phase in eukaryotes. The G-protein complex eIF2 associates with GTP and Met-tRNA_i_^Met^ to form the ternary complex (eIF2-TC) that, with the help of eIF1, eIF1A, eIF5 and the multisubunit complex eIF3, is delivered to the small ribosomal subunit to form the 43S PIC (reviewed in Valášek 2012; Hinnebusch 2017) (Fig. 1). The eIF4E (mRNA cap-binding protein), eIF4G (a scaffold protein) and eIF4A (a DEAD-box helicase), together comprising the eIF4F complex, bind to the mRNA 5^΄^ 7-methylguanosine cap and thus mediate mRNA recruitment to the 43S PIC to form the 48S PIC. Subsequently, the eIF4F complex, in conjunction with other RNA helicases (Ded1/Ddx3 in yeast; DHX29 in mammals), resolves secondary structures in the mRNA leader sequences to facilitate ribosomal scanning that occurs in the 5^΄^ to 3^΄^ direction until the AUG start codon has been recognized by base pairing with the anticodon of Met-tRNA_i_^Met^ (Hinnebusch 2014). According to the ‘first AUG rule’, for most mRNAs, the first AUG codon encountered by the scanning complex is favored to be selected as the initiation codon of the ORF, and the surrounding nucleotide sequences together with eIFs 1, 1A, 2, 3 and 5 modulate the efficiency of its selection (Hinnebusch 2017). Upon AUG selection and irreversible, eIF5-stimulated GTP hydrolysis on eIF2 (Algire, Maag and Lorsch 2005), the eIF2-GDP-eIF5 complex is released from the 48S PIC together with most other eIFs (reviewed in Jennings and Pavitt 2014; Dever, Kinzy and Pavitt 2016), except for eIF3 (Szamecz *et al.*^2008^; Mohammad *et al.*^2017^; Valasek *et al.*^2017^) and probably also eIF4F (Pöyry, Kaminski and Jackson 2004). eIF5B subsequently catalyzes the joining of the 60S subunit, and upon GTP hydrolysis on eIF5B and its release from the ribosome (together with eIF1A) (Dever, Kinzy and Pavitt 2016), the 80S initiation complex thus formed can enter the elongation phase. Protein synthesis then proceeds until a stop codon enters the ribosomal A-site and is recognized by the complex of eukaryotic release factors eRF1 and eRF3 (Fig. 1). eRF1, with the help of the recycling factor ABCE1 (Rli1 in yeast), catalyzes the hydrolysis of the peptidyl-tRNA in the ribosomal P-site, releasing the completed polypeptide and producing the 80S post-termination complex (80S post-TC) consisting of the 80S ribosome bound to mRNA with deacylated tRNA base paired to the penultimate codon in the ribosomal P-site (Dever and Green 2012; Jackson, Hellen and Pestova 2012). ABCE1, perhaps together with eRF1, then initiates the recycling phase by splitting the subunits and releasing the 60S subunit from the remaining 40S post-TC (Pisarev *et al.*^2010^) (Fig. 1). It was shown *in vitro* that the ejection of the P-site tRNA and release of the 40S subunit from the mRNA can be achieved either by the combined action of canonical initiation factors eIF1, eIF1A, eIF3 and the eIF3-associated factor eIF3j (Hcr1 in yeast) or alternatively by a non-canonical initiation factor eIF2D (also known as Ligatin) or the DENR-MCT-1 complex composed of two proteins that are homologous to the N- and C-terminal regions of eIF2D, respectively (Pisarev, Hellen and Pestova 2007; Skabkin *et al.*^2010^) (Fig. 1). The recycling phase thus ensures that at the end of each cycle, liberated mRNA and ribosomal subunits can immediately enter a new round of translation, which conserves energy and enables rapid regulatory responses when the demand for the synthesis of all or just a particular set of proteins changes.

Although ribosomal recycling is the natural sequence of events following translation termination, there are specific exceptions when the termination phase is followed by a second initiation event—reinitiation (REI)—on the same mRNA molecule at a site downstream (or in some cases even upstream) of the stop codon. This can be achieved either by (i) incomplete post-TC recycling, particularly by allowing only dissociation of the 60S subunit by ABCE1, presumably followed by release of the deacylated tRNA from the P-site of the 40S post-TC to allow REI via the small subunit in a manner similar to that of canonical initiation, or (ii) blocking the whole recycling process, in which case REI occurs with 80S post-TCs in a manner that may or may not require release of the deacylated tRNA (Fig. 1). One consequence of REI is the increase of the coding capacity of the genome and production of multiple proteins at calibrated levels from a single mRNA. Not surprisingly, therefore, REI occurs widely in organisms with tightly packed genomes such as prokaryotes and viruses. In some instances, however, the production of the upstream peptide is not physiologically important (especially when it is very short) and translation of the first ORF merely provides the means of controlling the translation rate of the main ORF downstream. REI in eukaryotic cells is considered to be less common than in prokaryotes and viruses since the majority of bacterial and viral transcripts are polycistronic, whereas nearly all eukaryotic mRNAs contain only a single long-coding sequence and REI, when it occurs, follows translation of relatively short upstream ORFs (uORFs). Nevertheless, the fact that REI events frequently occur on mRNAs encoding key regulatory proteins in response to specific internal or external stimuli (e.g. during various stresses) underscores the physiological importance of this translational control mechanism in enabling cells to adapt to changing environmental conditions.

In this review, we describe all known types of translation REI, some of which have been identified only very recently. According to the criteria described in detail below, we divide them into three main categories: (i) REI after short uORFs, which in the extreme case contain only an AUG and stop codon (‘start-stop’ uORFs), (ii) REI after long uORFs, and (iii) REI occurring within coding regions (a schematic of different forms of uORFs is shown in Fig. S1, Supporting Information). We summarize our current knowledge of molecular details of these REI mechanisms obtained from studies of various model organisms, including bacteria, yeast, plants, fruit fly and mammalian cells, as well as different fungal, plant, animal and human viruses, focusing on both *cis*-acting regulatory mRNA sequences and *trans*-acting regulatory proteins that have been discovered. Although it might seem at first glance that the different types of REI are largely unrelated mechanistically, many common features and principles are revealed through an in-depth analysis of the available information. In addition, we raise several questions that may spur future investigations and progress in this interesting field.

## REI AFTER TRANSLATION OF SHORT uORFs

REI after short uORFs arguably represents the most widely recognized type. One reason is that the occurrence of short uORFs is relatively widespread across both viral and eukaryotic genomes, for the latter being found in ∼13%, 35% ∼65%, ∼44% and ∼49% of yeast, plants, zebrafish, mouse and human transcripts, respectively (Calvo, Pagliarini and Mootha 2009; Lawless *et al.*^2009^; von Arnim, Jia and Vaughn 2014; Chew, Pauli and Schier 2016). Due to their minimal length, short uORFs are generally considered as merely regulatory elements governing expression of main ORFs without any significant coding potential. Taking into account the nature of the scanning mechanism for start codon selection described above, uORFs should in principle pose a functional barrier for translation of a downstream cistron and, indeed, most uORFs effectively downregulate expression of the main ORFs (Calvo, Pagliarini and Mootha 2009; Barbosa, Peixeiro and Romao 2013; von Arnim, Jia and Vaughn 2014; Wethmar 2014; Hinnebusch, Ivanov and Sonenberg 2016). Interestingly, uORFs are frequently found in certain classes of mRNAs with temporal or tissue-specific expression, or whose encoded proteins have dose-dependent functions, e.g. proto-oncogenes or other regulatory factors involved in differentiation, cell cycle, stress response, learning and memory formation (Calvo, Pagliarini and Mootha 2009; Barbosa, Peixeiro and Romao 2013; von Arnim, Jia and Vaughn 2014; Wethmar 2014; Janich *et al.*^2015^; Hinnebusch, Ivanov and Sonenberg 2016), which are upregulated only upon specific internal or external signals. Hence, it may not be surprising that deregulation of uORF translation and uORF polymorphisms have been implicated in a variety of human diseases (Calvo, Pagliarini and Mootha 2009; Barbosa, Peixeiro and Romao 2013; Wethmar 2014). Observations that near-cognate triplets (e.g. CUG, UUG, and GUG) can serve in addition to AUG as authentic initiation sites of short uORFs likely also contributes to the breadth of uORF-mediated translational control (Ingolia, Lareau and Weissman 2011; Fijalkowska *et al.*^2017^).

It is important to emphasize, however, that REI is not the only mechanism by which short uORFs control the expression of a downstream gene; others include increased uORF-triggered mRNA decay via the nonsense-mediated decay (NMD) pathway; constitutive or modulated ‘leaky scanning’, wherein the uORF start codon is bypassed to some degree by the scanning PIC; and regulated translational arrest or stalling within uORFs that modulates the proportion of scanning PICs able to reach the downstream ORF, for example, in response to an availability of a specific metabolite (for review, see Barbosa, Peixeiro and Romao 2013; Wethmar 2014; Hinnebusch, Ivanov and Sonenberg 2016). In fact, there are many cases, especially in transcripts of higher eukaryotes with multiple uORFs, where expression of the main ORF is regulated in a more complex way by combining two or more of these mechanisms (Barbosa, Peixeiro and Romao 2013; Wethmar 2014; Hinnebusch, Ivanov and Sonenberg 2016).

By definition, a short uORF is an open reading frame occurring in the 5^΄^ leader of a long ORF-containing mRNA. It is composed of a start codon and an in-frame termination codon separated by at least one additional sense codon. The ‘start-stop uORFs’, lacking even a single additional sense codon, have also been classified as canonical uORFs; however, since there is no elongation involved, they likely represent a separate functional class. In any case, the ability of an uORF to promote 40S-mediated REI (its REI-permissiveness) generally depends on four main factors: (i) *cis*-acting mRNA features surrounding the uORF; (ii) duration of uORF elongation, which is determined by its length and the propensity of its sequence to form stable secondary structures; (iii) a subset of initiation factors involved in primary initiation at the uORF start codon; and (iv) the intercistronic distance between the uORF and main ORF. This last parameter determines the probability that the 40S subunit acquires a new eIF2-TC while traversing the leader in the 5^΄^ to 3^΄^ direction before reaching the start codon of the downstream ORF, thus enabling recognition of the main ORF start codon by the anticodon of Met-tRNA_i_^Met^. Modulating the availability of the eIF2-TC also affects this probability. (Prior to eIF2-TC acquisition, the 40S subunit has no means to recognize the next AUG codon and, hence, this initial part of its journey will be referred to as ‘traversing’ rather than ‘scanning’.) (Kozak 1987; Pöyry, Kaminski and Jackson 2004; Szamecz *et al.*^2008^; Cuchalová *et al.*^2010^; Roy *et al.*^2010^; Munzarová *et al.*^2011^; Mohammad *et al.*^2017^). According to the aforementioned criteria for efficient REI, uORFs that are too long or overlap the main ORF are expected to be REI non-permissive. Indeed, studies indicate that REI-permissive uORFs are usually less than 10 codons in yeast, 16 codons in plants and 30 codons in mammals (Kozak 2001; Calvo, Pagliarini and Mootha 2009; von Arnim, Jia and Vaughn 2014).

Besides eIF2-TC, other eIFs, including eIF1 and 1A, are probably reacquired either at the onset of traversing or as the 40S traverses/scans further downstream from the uORF to promote proper recognition of the next start codon. It is well established that eIF1 restricts recognition of near-cognate start codons, and AUG start codons in poor Kozak context, and that changing the cellular availability of eIF1 alters the frequency of initiation events at such suboptimal start sites (Hinnebusch 2017; Ivanov *et al.*^2017^). Recent genome-wide analysis demonstrated that eIF1 depletion in mammalian cells evoked upregulation of uORFs with suboptimal starts. Consequently, uORFs that acted as poor barriers of the main ORF under normal conditions became more prominent barriers at reduced eIF1 levels (Fijalkowska *et al.*^2017^). By extension, the efficiency of REI on main ORFs with suboptimal start sites might increase at reduced eIF1 levels.

It is generally considered that the eIF2-TC and other eIFs that bind at the subunit interface of the 40S subunit (eIF1, eIF1A), and are released on 60S joining, must be reacquired from the cytosol after uORF termination. However, there is a long-standing hypothesis that certain eIFs important for REI remain at least transiently associated with the elongating ribosome, and that increasing the uORF length or time required for its translation increases the likelihood that these eIFs dissociate before the completion of uORF translation (Kozak 2001). When uORF translation takes a shorter period of time, these eIFs are still present at uORF termination and remain associated with the 40S post-TC following recycling of the 60S subunit, ready to enhance REI. So far, this phenomenon has been directly demonstrated *in vivo* only for yeast eIF3 (Mohammad *et al.*^2017^), in accordance with the fact that it binds primarily to the surface of the 40S subunit that remains solvent-exposed in the translating 80S ribosome (Valášek *et al.*^2003^; Hashem *et al.*^2013^; Aylett *et al.*^2015^; des Georges *et al.*^2015^; Llacer *et al.*^2015^) (Fig. 1 and Fig. S2, Supporting Information). However, genetic experiments suggest that participation of eIF3 in REI is also conserved in higher eukaryotes (Roy *et al.*^2010^; Hronova *et al.*^2017^). Studies in mammalian reconstituted systems indicate that eIF4F might also persist transiently on elongating ribosomes and thereby facilitate REI following translation of short uORFs (Pöyry, Kaminski and Jackson 2004; Skabkin *et al.*^2013^). eIF4F might function generally by opening the mRNA entry channel of the 40S ribosome that is traversing downstream, or it may be critical especially for REI events where the 40S subunit must traverse/scan sequences burdened with secondary structures that can be unwound by eIF4F (Sen *et al.*^2015^). Besides canonical eIFs, some short uORFs seem to utilize other, REI-specific *trans*-acting factors as well (see below).

### Regulation of REI on short uORF(s)-containing mRNAs by eIF2α phosphorylation

#### REI on *GCN4* in yeast and other fungi

**Figure 2.**
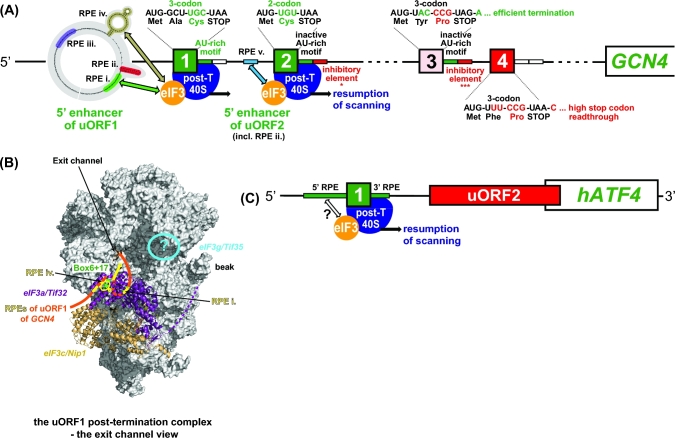
(**A**) Model of the 5^΄^ leader of *GCN4* mRNA with its four short uORFs summarizing all REI-promoting and inhibiting RNA and protein features (adapted from Gunisova *et al.*^2016^). For details, see the main text. (**B**) Graphical illustration of the proposed arrangement of the post-termination complex on uORF1 with its RPEs interacting with Box 6 and Box 17 segments of the N-terminal domain of a/Tif32 to promote resumption of scanning for REI on *GCN4* (adopted from Mohammad *et al.*^2017^). The exit channel view of the 48S closed complex shows only two incomplete eIF3 subunits for simplicity: eIF3c/Nip1 in light gold and eIF3a/Tif32 in purple, with its CTD represented by a dotted line (the structure of this domain is unknown and thus its placement in the 48S complex was only predicted). The location of Boxes 6 + 17 is indicated in green. The 5^΄^ leader of uORF1 is shown in orange with its RPEs depicted in yellow. The predicted position of eIF3g/Tif35 is indicated by the blue circle. (**C**) Model of the *ATF4* mRNA; RPEs surrounding uORF1 are depicted in green and the prospective interaction between eIF3 and the 5´ RPE is indicated. For details, see the main text.

Undoubtedly, the best described example of regulation of REI on short uORF-containing mRNAs by phosphorylation of the α-subunit of eIF2 (eIF2α) in response to nutritional stress is the *Saccharomyces cerevisiae GCN4* gene (Fig. 2A). *GCN4* encodes a master, basic leucine zipper (bZIP) transcription factor that activates, among many others, amino acid biosynthetic genes in response to amino acid limitation in the so-called general amino acid control (GAAC) pathway (reviewed in Hinnebusch 2005). Because eIF2α phosphorylation downregulates bulk translation, this regulatory response enables cells to swiftly limit consumption of amino acids by general protein synthesis while allowing their usage for inducing the synthesis of Gcn4 and ∼600 other stress-response proteins under Gcn4 control, thereby increasing amino acid availability under conditions of amino acid scarcity.

The *GCN4* mRNA leader contains four short AUG-initiated uORFs with a relatively close spacing between uORFs 1 and 2, even closer spacing between uORFs 3 and 4, and relatively larger separations between uORFs 2 and 3, and uORF4 and the *GCN4* ORF (Fig. 2A). Most of the experimental evidence establishing the mechanism of *GCN4* translational control is genetic and involves an extensive panel of mutations that systematically alter the individual uORFs or their surrounding sequences. The outcome on REI efficiency is measured by a reporter with the *GCN4* main ORF fused to *lacZ* and compares expression of wild type (WT) versus mutant reporter constructs. It should be stressed that early studies justified the use of this reporter by confirming that expression of various mutant *GCN4-lacZ* reporters paralleled the expression of particular Gcn4 target genes (or cellular phenotypes dependent on the GAAC response) in strains harboring the corresponding mutant *GCN4* alleles on single-copy plasmids (Mueller and Hinnebusch 1986; Mueller *et al.*^1988^; Miller and Hinnebusch 1989; Grant and Hinnebusch 1994; Grant, Miller and Hinnebusch 1995).

The basic principles of the delayed REI mechanism on the *GCN4* mRNA can be best described using a simplified model featuring only uORFs 1 and 4 (Fig. S3, Supporting Information), as described in Hinnebusch (2005). The 5^΄^ proximal uORF1 is a positive, REI-promoting feature required for induction of *GCN4* in starved cells, while the 5^΄^ distal uORF4 is a negative, REI-suppressing feature required for *GCN4* repression in non-starved cells. Whereas uORF1 is highly permissive for REI, uORF4 is non-permissive, such that for *GCN4* translation to occur, REI at uORF4 must be avoided. This is achieved by delaying the acquisition of eIF2-TC by 40S subunits traversing the *GCN4* leader following translation of uORF1, so that a fraction of subunits arrive at uORF4 without the eIF2-TC, and thus unable to recognize the AUG codon at this uORF. They acquire the eIF2-TC only after bypassing uORF4 and so can reinitiate at *GCN4* instead. The key evidence for the importance of the delayed acquisition of the eIF2-TC came from progressively increasing the uORF1-uORF4 distance resulting in gradual decline in REI efficiency at the *GCN4* AUG (Abastado *et al.*^1991^). The delayed acquisition of the eIF2-TC is achieved in amino acid-starved cells. Amino acid deficiency is sensed by the Gcn2 kinase that phosphorylates eIF2 on Ser51 of its alpha subunit and thus effectively prevents *de novo* formation of the eIF2-TC complexes. This stress response shuts down general translation initiation, as eIF2-TCs are required for translation of most mRNAs, but at the same time stimulates *GCN4* mRNA translation (reviewed in Hinnebusch 2005; Jennings *et al.*^2017^).

**Figure 3.**
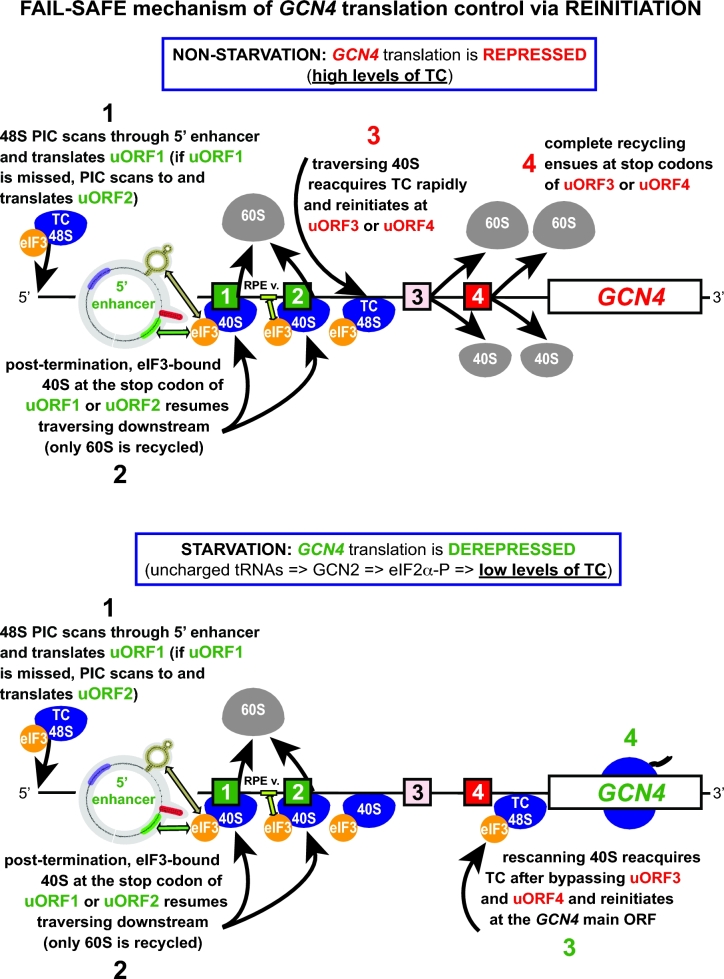
Model for *GCN4* translational control under non-starvation versus starvation conditions mediated via reinitiation in response to changing levels of the eIF2-TC. For details, see the main text (adapted from Gunisova and Valasek 2014).

Returning to the native *GCN4* mRNA containing all four uORFs, a comparison of individual REI efficiencies for each of the uORFs, determined with *GCN4-lacZ* constructs harboring each solitary uORF, showed that their propensities to promote REI markedly differ. uORF1 and uORF2 are the most REI-permissive, allowing approximately one-third of the *GCN4-lacZ* expression observed in the absence of all four uORFs (Mueller and Hinnebusch 1986). Normalizing the REI efficiency of uORF1 to 100%, uORF2 is ∼90% efficient, whereas uORF3 and uORF4 are largely REI non-permissive, enabling only ∼18% and ∼4% REI, respectively (Munzarová *et al.*^2011^; Gunisova and Valasek 2014). As outlined below (Fig. 3), this arrangement of one pair of REI-permissive uORFs followed by another pair of REI–non-permissive uORFs creates a ‘fail-safe’ mechanism that ensures maximal *GCN4* induction under starvation conditions and, at the same time, tight inhibition of its expression under non-starvation conditions (Munzarová *et al.*^2011^; Gunisova and Valasek 2014).

The ability of *GCN4* uORFs to allow or prevent REI is determined by several *cis*-acting elements. Sequences upstream of uORF1 are required for its high REI potential (Grant, Miller and Hinnebusch 1995) and multiple REI-promoting elements (RPEs) have been mapped upstream of both uORF1 and uORF2 that create a specific structural arrangement (the 5^΄^ enhancer) upstream of these two ORFs (Fig. 2A) (Munzarová *et al.*^2011^; Gunisova and Valasek 2014). uORF1 utilizes four RPEs (i–iv), whereas uORF2 separately utilizes only a single RPE (v, with sequence similarity to RPE i), and, in addition, shares RPE ii with uORF1 (Fig. 2A). A combination of computational analysis and enzymatic probing showed that the shared RPE ii forms a stable stem loop, whereas RPE iv assembles into a double-circle hairpin (Munzarová *et al.*^2011^; Gunisova and Valasek 2014) (Fig. 2A).

Besides the RPEs, two separate regions were identified within the extreme N-terminal domain (NTD) of the a/Tif32 subunit of eIF3 (called Box 6 and Box 17) that proved to be critical in *trans* for the high REI competence of these two uORFs (Fig. 2B) (Szamecz *et al.*^2008^; Munzarová *et al.*^2011^). Genetic epistatic experiments revealed that RPEs i and iv of uORF1 and RPE v of uORF2 cooperate with these two segments of a/Tif32 to promote efficient REI (Munzarová *et al.*^2011^; Gunisova and Valasek 2014). Importantly, the a/Tif32-NTD has a favorable location on the 40S subunit next to the mRNA exit channel (Valášek *et al.*^2003^; Kouba *et al.*^2012^; Aylett *et al.*^2015^), where it could directly contact these RPEs, which during termination on uORF1 or uORF2 will be emerging from the mRNA exit channel (Fig. 2B). This could stabilize the 40S post-TC, particularly following dissociation of the deacylated tRNA cognate to the penultimate codon of the uORF (more on this below), to allow resumption of traversing/scanning downstream. A structural motif similar to RPE iv was also identified upstream of the REI-permissive uORF in the mRNA leader of another yeast transcriptional activator Yap1. The fact that it likewise operates in an a/Tif32-NTD-dependent manner (Munzarová *et al.*^2011^) suggests that, at least in yeasts, the underlying molecular mechanism of REI on short uORFs might be conserved.

Based on these findings, we proposed that while the eIF3-bound 40S ribosome scans through the region upstream of uORF1 (or uORF2) and subsequently translates one of these uORFs—retaining eIF3 during elongation, the RPEs progressively fold into the aforementioned secondary structures. Upon termination and dissociation of the 60S subunit, eIF3 interacts with the corresponding RPEs to specifically stabilize the 40S post-TC at the uORF1 (or uORF2) stop codon (Fig. 2B). Upon dissociation of the P-site-bound deacylated tRNA and acquisition of other eIFs like eIF1 and eIF1A that might be needed for the subsequent step, the 40S subunit will resume traversing downstream. Once it reacquires the eIF2-TC, it switches into the scanning mode and begins ‘searching’ for the next start codon. Employing a novel *in vivo* RNA–protein Ni^2+^ pull-down (RaP-NiP) assay, we provided direct *in vivo* evidence that eIF3 indeed remains transiently bound to elongating ribosomes post-initiation and interacts with the *cis*-acting elements of uORFs 1 and 2 (Mohammad *et al.*^2017^). More experiments are needed to reveal whether the cooperation between the a/Tif32-NTD and RPEs in rendering uORFs 1 and 2 REI-competent stems from a direct contact between them as opposed to an indirect functional interaction. Patches of positive charge on the surface of the a/Tif32-NTD and its ability to bind RNA (Khoshnevis *et al.*^2014^) may favor the model of direct contact. Molecular roles of RPE ii and iii, which function independently of eIF3 (Munzarová *et al.*^2011^; Gunisova and Valasek 2014), remain to be determined, as does the importance of eIF4F in REI, in view of its predicted ability to remain associated with the ribosome during early elongation (Pöyry, Kaminski and Jackson 2004; Skabkin *et al.*^2013^). Besides eIF3a, the eIF3g/Tif35 subunit also stimulates REI after translation of both REI-permissive uORFs (Cuchalová *et al.*^2010^; S.G. and L.S.V., unpublished data). Based on its interactions with Rps3/uS3 and Rps20/uS10 (Cuchalová *et al.*^2010^), g/Tif35 appears to reside near the mRNA entry channel (Fig. 2B) (Aylett *et al.*^2015^); however, the molecular basis of its contribution to REI is unknown.

In contrast to uORF2, the REI competence of uORF1 additionally depends on the AU-rich nature of sequences immediately following its stop codon, and replacing these sequences with the corresponding sequences from uORF4 impaired REI (Miller and Hinnebusch 1989; Grant and Hinnebusch 1994). The AU-rich motif identified within the first 12 nt of the uORF1 3^΄^ sequences was recently shown to be critical for REI (Gunisova *et al.*^2016^) (Fig. 2A). Although nearly the same motifs occur in the 3^΄^ sequences of uORFs 2 and 3, they do not promote REI of these two uORFs. In fact, the AU-rich motif operates only at uORF1, independently of the RPEs, in a manner strictly dependent on its position following the uORF1 stop codon, and is an essential prerequisite for the function of the 5^΄^ enhancer at uORF1 (Munzarová *et al.*^2011^; Gunisova *et al.*^2016^). However, the molecular mechanism of the AU-rich element in uORF1 REI is unknown. Considering its position-specific role and the fact that it will be buried in an 80S ribosome terminating at uORF1, it might function either to speed up recycling of the 60S subunit and/or deacylated tRNA or to prevent recycling of the post-termination 40S subunit, perhaps by a looping interaction with more remote sequences within the *GCN4* mRNA leader. Intriguingly, besides the REI-promoting sequences, REI-inhibiting sequences were found in more distal 3^΄^ regions of uORF2 and uORF3 following their respective AU-rich motifs (Fig. 2A). These sequences function irrespectively of their distance from the *GCN4* start codon and decrease to some extent the REI potential of these two uORFs (Gunisova *et al.*^2016^), presumably to optimize the dynamic range of *GCN4* translational control. However, the molecular details of their inhibitory functions are also unknown.

The REI potential of *GCN4* uORFs is further modulated by their coding sequences, mainly by the character of the last sense codons (Miller and Hinnebusch 1989; Grant and Hinnebusch 1994; Gunisova *et al.*^2016^). At uORF1 and uORF2, UGC and UGU cysteine codons are found, respectively, while uORF3 and uORF4 both contain the CCG proline codon at that position (Fig. 2A). The presence of Cys and Pro codons as the last coding triplets of the short uORFs in *GCN4* mRNA is conserved in yeast species related to *S. cerevisiae*. Intriguingly, tRNA^Cys^ was shown to be particularly prone to spontaneous dissociation from the ribosomal P-site in post-TCs analyzed *in vitro* (Skabkin *et al.*^2013^). Hence, the presence of Cys as the last sense codon at uORFs 1 and 2 might facilitate stabilization of the post-TC 40S subunits, as spontaneous dissociation of deacylated tRNA^Cys^ could eliminate the need for the ‘second-stage’ recycling factors, eIF1 and eIF1A or eIF2D to catalyze its removal. Rapid, stochastic dissociation of deacylated tRNA^Cys^ following 60S subunit recycling, combined with the known role of eIF3 in mRNA stabilization on the ribosome (Kolupaeva *et al.*^2005^; Jivotovskaya *et al.*^2006^; Khoshnevis *et al.*^2014^; Aitken *et al.*^2016^), presumably via a/Tif32-NTD interactions with RPEs i and iv of uORF1 or RPE v of uORF2 (Fig. 2B), could thus prevent full ribosomal recycling and allow the post-TC 40S subunit to resume traversing downstream. The ability of the conserved CCG Pro codons to suppress the REI potential of uORF3 and uORF4 might be attributable to the spatially restricted conformation of the proline residue, which could prevent efficient REI either by slowing down the speed of uORF3 and 4 translation (Wohlgemuth *et al.*^2008^; Pavlov *et al.*^2009^) or by interfering with stop codon recognition in translation termination. Interestingly, the ∼4-fold difference between the REI potential of uORF3 and uORF4 is largely determined by their different second codons and stop codon tetranucleotides (i.e. the stop codon plus the immediately following base), which at uORF4 further diminishes its REI potential by allowing a higher frequency of stop codon readthrough (Beznoskova, Gunisova and Valasek 2016; Gunisova *et al.*^2016^). This prolongs the elongation phase of uORF4 translation by additional 22 codons before the next in-frame stop codon is encountered with attendant reduction in REI potential.

Taken all together, the complex translational regulation of *GCN4* under nutrient replete versus depleted conditions ultimately reflects the differential translation of all four uORFs that, according to their REI properties, control the fate of ribosomes terminating at their stop codons. The key real ‘decision makers’ are the REI–non-permissive uORFs 3 and 4, whose expression prevents *GCN4* to be translated, whereas skipping these uORFs allows it. The relatively high REI potential of uORF2 provides a ‘fail-safe’ mechanism for *GCN4* translational control. As summarized in Fig. 3, the REI-permissive uORF1 is efficiently translated under both nutrient-replete and depleted conditions. After its translation, the post-TC 40S subunit remains bound to the mRNA with the help of eIF3 and resumes traversing downstream. uORF2 serves as a backup for uORF1 to capture ribosomes that leaky-scanned past uORF1’s AUG, thereby maximizing the REI potential of the whole system. This could be especially important during stress, where the frequency of leaky scanning appears to be elevated (Baird and Wek 2012; Barbosa, Peixeiro and Romao 2013). In non-starved cells, where the eIF2-TC levels are high, nearly all of the 5^΄^ to 3^΄^-migrating ribosomes rebind the eIF2-TC before reaching uORF3 or uORF4. Since neither of these uORFs supports efficient REI, scanning ribosomes that reinitiate there will undergo full ribosomal recycling following termination, thus preventing REI at *GCN4* (Fig. 3)*.* Under starvation conditions characterized by low levels of the eIF2-TC, a large proportion of the post-termination 40S ribosomes will bypass uORFs 3 and 4 and upon eventual acquisition of the eIF2-TC reinitiate at the *GCN4* start codon (Fig. 3). It is worth mentioning that besides the extensive genetic evidence supporting this model, key tenets of the mechanism were supported by analysis of ribosome-protected mRNA fragments by toe-printing and ribosome profiling techniques (Gaba *et al.*^2001^; Ingolia *et al.*^2009^).

Homologs of *GCN4* in filamentous fungi contain minimally two uORFs, and it is generally assumed that they perform similar functions as uORF1 and uORF4 of *S. cerevisiae GCN4.* This assumption finds strong support in observations that each of the two REI-permissive uORFs in combination with either of the two REI–non-permissive uORFs suffice for the qualitative aspects of *GCN4* translational control in *S. cerevisiae* (Mueller and Hinnebusch 1986; Gunisova and Valasek 2014), as mentioned above. The best-studied homolog in filamentous fungi is *cpc-1* of *Neurospora crassa*, whose translation is induced in starvation conditions through eIF2α phosphorylation by the Gcn2 homolog encoded by *cpc-3*. Induction is dependent on two uORFs, which orchestrate a cross-pathway control (CPC) response analogous to GAAC (Luo *et al.*^1995^; Sattlegger, Hinnebusch and Barthelmess 1998). Recently, evidence was presented that post-termination 40S ribosomes efficiently reinitiate after translation of the 3-codon long uORF1 but not after translation of much longer uORF2 in the *cpc-1* mRNA leader (Ivanov *et al.*^2017^), consistent with the *GCN4* mechanism. However, whether there are other mechanistic parallels among the RPEs of *S. cerevisiae GCN4* uORF1 and its counterpart in *N. crassa cpc-1* remains to be seen.

Unexpectedly, in the pathogenic yeast *Candida albicans*, a different means of translational control not involving REI appears to regulate *GCN4* expression. Although the 5^΄^ leader of *C. albicans GCN4* also contains multiple uORFs (three in total), in contrast to *S. cerevisiae* and *N. crassa GCN4/cpc-1*, uORF3 alone is sufficient for translational regulation. Under non-stress conditions, uORF3 inhibits *GCN4* translation. Amino acid starvation conditions promote Gcn2-mediated phosphorylation of eIF2α and leaky ribosomal scanning to allow bypass of uORF3 and translation of the *GCN4* main ORF instead, inducing *GCN4* expression. It was suggested that it is particularly important that Gcn4 levels are tightly controlled since Gcn4 regulates morphogenetic changes during amino acid starvation conditions, which are important determinants of virulence in this fungus (Sundaram and Grant 2014).

#### REI on *ATF4* in mammals

*GCN4* has also a functional homolog in mammalian genomes, *ATF4* (activating transcription factor 4), that, like *cpc-1*, contains a 3-codon uORF1 and a longer uORF2, which in this case overlaps the beginning of the main ORF (Fig. 2C). According to the *GCN4* model, phosphorylation of mammalian eIF2α induces *ATF4* translation (Lu, Harding and Ron 2004; Vattem and Wek 2004), which can be achieved in principle by activation of any of four different mammalian eIF2α kinases: GCN2, PERK, PKR and HRI1 (Pakos-Zebrucka *et al.*^2016^). Each of these kinases is activated by a different type of stress and their functions converge in the so-called integrated stress response (ISR) (Pakos-Zebrucka *et al.*^2016^) (note that budding yeasts contain only Gcn2).

Experiments with mouse reporter constructs revealed that, similar to *GCN4* uORFs 1 and 4*, ATF4* uORF1 is a positive, stimulatory feature allowing efficient REI after its translation, whereas translation of uORF2 inhibits *ATF4* expression, and translation of uORF1 combined with low levels of the eIF2-TC are required to overcome the uORF2 inhibitory effect (Lu, Harding and Ron 2004; Vattem and Wek 2004). It was recently demonstrated that, by analogy with *GCN4* uORF1, *ATF4* uORF1 is surrounded by *cis*-acting, REI-promoting sequences, with the upstream sequences most probably forming specific secondary structures (Hronova *et al.*^2017^) (Fig. 2C). In addition, it was shown that efficient REI at the human *ATF4* main ORF requires the eIF3h subunit, previously implicated in REI in plants (see below). Although it is not known whether eIF3h functionally cooperates with sequences upstream of *ATF4* uORF1, like eIF3a/Tif32 does in yeast, it seems likely that the basic molecular principles of REI are conserved between yeast *GCN4* and human *ATF4* (Fig. 2C).

Despite the evidence for translational control via uORF1-mediated REI underlying stress-induced ATF4 synthesis (Lu, Harding and Ron 2004; Vattem and Wek 2004), many recent studies indicate that other outputs of phosphorylated eIF2α specific to higher eukaryotes, or even other regulatory pathways unrelated to the ISR, make important contributions to ATF4 induction. Various stresses were shown to stimulate *ATF4* transcription (see for example Dey *et al.*^2010^), increase the stability of *ATF4* mRNA by inhibition of NMD (Gardner 2008), increase the level of ATF4 protein by preventing its degradation (see for example Koditz *et al.*^2007^), or boost *ATF4* translation by other mechanisms besides REI such as by leaky scanning (Starck *et al.*^2016^). In fact, in direct contradiction with the original model, three independent studies noted increased translation of uORF2 under stress conditions (Andreev *et al.*2015a; Sidrauski *et al.*^2015^; Starck *et al.*^2016^).

While there is little doubt that translational upregulation of *ATF4* during stress is achieved partly by uORF1-mediated REI, the contribution of this mechanism to the overall increase in ATF4 protein levels might be only ∼2-fold vs the ∼6-fold originally reported (Vattem and Wek 2004; Andreev *et al*. 2015a; V.H. and L.S.V. unpublished data). It should be noted that even in yeasts, amino acid starvation increases the level of *GCN4* mRNA ∼2-fold and the rate of Gcn4 degradation by the proteasome is also diminished under conditions of severe starvation, augmenting the translational induction of *GCN4* at different levels of gene expression (Hinnebusch 2005). There are several other mammalian examples of the short uORF-mediated REI mechanisms regulating expression of *C/EBPα* and *C/EBPβ*, *CD36* and *ELK-1* that we will not discuss in detail (Calkhoven, Muller and Leutz 2000; Griffin *et al.*^2001^; Rahim *et al.*^2012^). It suffices to say that translational control of these genes is also not mediated solely by REI but by a combination of REI with other translation control mechanisms, including leaky scanning. In fact, both these mechanisms were recently observed operating on mRNAs by a detailed analysis of ribosomal profiling of a neural cell line under complete oxygen and glucose deprivation when the stringency of AUG start codon selection is significantly reduced (Andreev *et al*. 2015b). Taken altogether, it remains to be seen whether there are at least some mammalian genes whose expression is governed solely by REI. Perhaps the evolution of more complex organisms has necessitated more elaborate translational controls, such that the overall regulatory output is always a combination of multiple inputs.

### Regulation of REI of short uORF(s)-containing mRNAs by eIF3h phosphorylation in *Arabidopsis thaliana*

The uORF-mediated control of translation has also been found to play a key role in complex growth and developmental processes in plants. In *A. thaliana*, the mRNA leaders involved in such regulation typically contain multiple short or long uORFs that are frequently overlapping, which complicates the assessment of their contributions to REI efficiency. Well-studied examples of plant REI include the receptor for kinase Clavata 1 (CLV1), leaf transcription factor ASYMETRIC LEAVES 1 (AS1), as well as several members of two families of transcription factors, namely auxin response factors (ARFs) and bZIP factors. In all of these cases, the intact eIF3h subunit of eIF3 is required to overcome the inhibitory effect of uORFs to allow efficient REI at the main ORF of the respective gene (Nishimura *et al.*^2005^; Kim *et al.*^2007^; Roy *et al.*^2010^; Zhou, Roy and von Arnim 2014). Strong specificity for eIF3h function in these REI events was demonstrated by showing that a C-terminal truncation of eIF3h that reduces the subunit's association with the rest of eIF3 and the 43S PIC selectively decreased translation of the aforementioned main ORFs while showing no impact on global translation initiation rates (Kim *et al.*^2004^, ^2007^; Roy *et al.*^2010^). It was proposed that, by analogy with the function of yeast eIF3a/Tif32 discussed above, eIF3h (specifically its N-terminal part) might support efficient REI by preserving the competence of a fraction of uORF-translating ribosomes to resume traversing downstream (Roy *et al.*^2010^). Whether eIF3h stabilizes only eIF3 or mRNA, or both, on the post-termination 40S subunits, and whether it acts on its own or in cooperation with some other eIF3 subunits or other factors, remains to be determined. In any case, polysomal microarray analysis clearly demonstrated that eIF3h is a general stimulator of efficient translation of short uORF(s)-containing mRNAs throughout the transcriptome (Kim *et al.*^2007^).

**Figure 4.**
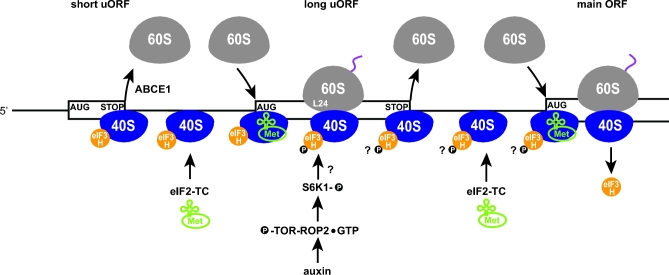
Model for translational control of short and long uORFs-containing mRNAs mediated via reinitiation promoted by eIF3h phosphorylation in *A. thaliana.* For details, see the main text.

Interestingly, the efficiency of REI on ARF-encoding mRNAs and also on the auxin-unrelated *AtbZIP11* mRNA can be further increased upon activation of the auxin signaling pathway. Recent data revealed that transduction of the signal into activation of translation requires coordinated action of phytohormone auxin, Rho-like small GTPase from plants 2 (ROP2), the central growth regulator serine/threonine protein kinase TOR (target of rapamycin) and eIF3h (Schepetilnikov *et al.*^2013^) (Fig. 4). Ribosome fractionation experiments indicated that, in response to auxin, polysomes show increased accumulation of uORF-containing mRNAs (indicating enhanced translation), phosphorylated and thus activated TOR, and, interestingly, phosphorylated eIF3h. On the other hand, the downstream effector of TOR, the 40S ribosomal protein S6 kinase 1 (S6K1), resides in polysomes mainly in its inactive form and its polysome association is disrupted immediately upon being phosphorylated by TOR (Schepetilnikov *et al.*^2013^). Supporting this, a similar mechanism was previously shown to operate in mammals, where activated mTOR phosphorylates eIF3-bound S6K1 in PICs, which triggers S6K1 activation and its subsequent dissociation from PICs (Holz *et al.*^2005^). The plant data thus may imply that PICs and polysomes serve as two relatively independent platforms for S6K1 activation via phosphorylation by TOR. Because plant eIF3h physically interacts with S6K1, it was further suggested that eIF3h might be the downstream target of activated S6K1 (Schepetilnikov *et al.*^2013^). It is theoretically possible that eIF3h phosphorylation is a trigger for S6K1 departure from polysomes. In summary, the current model proposes that the increased translation of main ORFs in short uORF(s)-containing mRNAs is triggered by activation of TOR by the GTP-bound ROP2 in response to auxin. Upregulated TOR is recruited to polysomes where it phosphorylates S6K1, which shortly before its release from polysomes phosphorylates eIF3h. This signaling cascade somehow ensures that ribosomes retain or adopt the REI-competent state to enable synthesis of the main ORF in response to auxin (Fig. 4).

In addition to eIF3h, the 60S ribosomal protein Rpl24/eL24 was also found to increase the REI competence of virtually the same classes of mRNAs (Fig. 4). Interestingly, mutations in either Rpl24/eL24 or eIF3h affect REI similarly and confer similar developmental defects, suggesting that the molecular functions of Rpl24/eL24 and eIF3h in plant REI are closely related (Nishimura *et al.*^2005^; Zhou, Roy and von Arnim 2010). However, the precise role of Rpl24/eL24 and the molecular details of the functional interaction between eIF3h and Rpl24/eL24 are unknown. Considering their distant locations on the ribosome, it is unlikely that they interact physically.

The key features just described for the auxin-stimulated REI mechanism, including hyperstimulation by a specific signal, involvement of TOR and ribosomal protein Rpl24/eL24, and the presumed requirement for retaining eIF3 during translation elongation, bear remarkable similarities with another REI mechanism found in *Cauliflower mosaic virus* (CaMV) that, by contrast, promotes REI after translation of long ORFs (Thiebeauld *et al.*^2009^; Schepetilnikov *et al.*^2011^) (for details, see below). What is the significance of having all these features in place to promote REI on mRNAs containing short or long uORFs, when the different uORF lengths should dictate distinct REI mechanisms? Perhaps, the answer lies in the fact that the leaders of the investigated mRNAs contain multiple uORFs and usually at least one of them is too long (often even longer than 90 codons) to allow the efficient short uORF-mediated REI defined above for *GCN4*. It is therefore conceivable that two types of REI mechanism operate together on the same mRNA bearing multiple uORFs of different lengths (Fig. 4). Whereas REI after short uORFs would rely on the four requirements established above, which apply to *GCN4*, REI after longer uORFs might require the signaling cascade that allows eIF3, via its h subunit, to persist longer on elongating ribosomes. Specific conformational changes of the ribosome mediated either directly or indirectly by ribosomal proteins, such as Rpl24/eL24, perhaps in response to signaling, may further buttress eIF3 retention. These thoughts find some support in reports showing that inhibition of TOR signaling did not affect translation of a reporter containing only a very short uORF, and that REI dependence on eIF3h was lost when the longest uORF (>40 codons) in the multiple uORF mRNA leader of *AtbZIP11* was removed (the remaining uORFs had a maximal length of only 20 codons) (Kim *et al.*^2007^; Schepetilnikov *et al.*^2013^).

### DENR-MCT-1 as REI-specific factors

**Figure 5.**
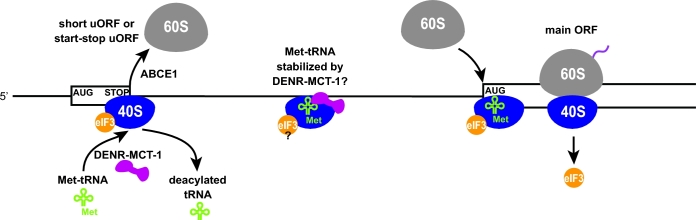
Model for translational control of short uORFs or start-stop uORFs-containing mRNAs mediated *via* reinitiation promoted by DENR-MCT-1 as the REI-specific factors. For details, see the main text.

The two subunits of the heterodimeric complex DENR-MCT-1 (MCTS1 in human) were identified as REI-specific *trans*-acting factors for certain short uORFs in *Drosophila* and humans (Schleich *et al.*^2014^, ^2017^). In *Drosophila*, DENR-MCT-1 was found to regulate a specific group of mRNAs possessing strong Kozak context at the AUG codons of their short uORFs; no additional *cis*-acting sequences seemed to be necessary (Fig. 5). The DENR-MCT-1 heterodimer promotes REI even in non-stressed, normally proliferating cells, i.e. when general translation is not compromised, and independently of the distance between an uORF and the main ORF (Schleich *et al.*^2014^). This implies that, unlike the *GCN4*-related mechanisms, REI seems to occur independently of the eIF2-TC abundance. Importantly, in contrast to eIF3 or eIF4F, the DENR-MCT-1 heterodimer is not required for initiation on uORF-less mRNAs (Schleich *et al.*^2014^), apparently uncoupling REI from standard initiation. However, the ability of DENR-MCT-1 to promote REI declines as uORFs become longer, suggesting that one or more eIFs from the primary initiation event are needed to ensure maximal REI. The uORF length requirements were even more strict in human cells, where, somewhat unexpectedly, DENR-MCT-1 supported REI only on reporter mRNAs bearing minimal ‘start-stop uORFs’ with AUGs in strong Kozak context (Schleich *et al.*^2017^). Interestingly, mRNA leaders of this type were found to be enriched in neuron-specific genes (Schleich *et al.*^2017^). These remarkable findings suggesting that REI-specific factors are not involved in canonical initiation raises many questions. Are there different types of short uORFs with markedly varying needs for auxiliary factors? It is possible that DENR-MCT-1-mediated REI can occur only in specific tissues or during restricted periods throughout development as a function of modulated expression of DENR or MCT1 proteins, or canonical eIFs.

It should be recalled that DENR-MCT-1 were shown *in vitro* to promote dissociation of deacylated tRNA and mRNA from the post-TC 40S subunits in the second step of ribosome recycling (Fig. 1) (Skabkin *et al.*^2010^). In contrast to removal of the deacylated tRNA from the P-site, the mRNA dissociation function of DENR-MCT-1 would be expected to inhibit rather than promote REI *in vivo*. DENR-MCT-1 were also shown *in vitro* to recruit Met-tRNA_i_^Met^ to the 40S subunit in a non-canonical, eIF2-independent manner on certain viral mRNAs that position the start codon directly in the P-site without any scanning (Skabkin *et al.*^2010^). This activity could have a stimulatory, rather than inhibitory, effect on REI. If DENR-MCT-1 exerts these functions in living cells, it would seem that the mRNA dissociation function would have to be inhibited in order to exploit the second function for Met-tRNA_i_^Met^ recruitment to stimulate REI. The question then is what determines whether DENR-MCT-1 completes the recycling reaction or promotes REI instead?

Since the ability of DENR-MCT-1 to promote REI decreases with increasing uORF length, it seems clear that the uORF length (and everything related to it) dictates the fate of the DENR-MCT-1-bound terminating ribosomes. This might favor the idea that DENR-MCT-1 cooperates with some factors that were involved in the primary initiation event and subsequently carried along with the elongating ribosomes for a limited number of elongation rounds—like eIF3 in yeast (Fig. 5).

Insights into DENR-MCT-1 functions were recently provided by two independent groups that resolved structures of human 40S complexes with DENR-MCT-1 or the related single polypeptide eIF2D (Lomakin *et al.*^2017^; Weisser *et al.*^2017^); one of which also contained mRNA with Met-tRNA_i_^Met^ based-paired with AUG in the P-site (Weisser *et al.*^2017^). The structures identified specific contacts of DENR-MCT-1 or eIF2D with the 40S subunit in the vicinity of the P-site, as well as contacts with both aminoacyl acceptor and anticodon arms of initiator tRNA that at least partially overlap with the known 40S-binding sites of eIF1, eIF2, and most probably also eIF1A. Interestingly, the positions of DENR-MCT-1 or eIF2D were also predicted to overlap with 40S contacts of specific domains of the eIF3a and eIF3b subunits known to transiently relocate to the 40S interface surface (Llacer *et al.*^2015^; Lomakin *et al.*^2017^; Valasek *et al.*^2017^; Weisser *et al.*^2017^) in contrast to the majority of eIF3 contacts that occur on the solvent-exposed side of the 40S (Fig. S2, Supporting Information). These latter eIF3 contacts, which presumably persist in the elongation complex (Mohammad *et al.*^2017^), could also co-exist with DENR-MCT-1 bound to the interface surface of the 40S. Hence, it is tempting to speculate that the persistence of eIF3 on elongating ribosomes selectively inhibits the mRNA recycling function of DENR-MCT-1 during termination on certain short uORFs in favor of REI (Fig. 5). The *in vitro* documented ability of DENR-MCT-1 to replace the deacylated tRNA in the P-site with Met-tRNA_i_^Met^, which gains support from multiple contacts of DENR-MCT-1 with Met-tRNA_i_^Met^ observed in the recent structure, coupled with the partial overlap of DENR-MCT-1 with the 40S-binding site of eIF2, could then eliminate the need of the post-termination 40S ribosome to rebind the eIF2-TC (Fig. 5), and thus might eliminate the requirement for a minimum intercistronic distance between the uORF and main ORF during REI. In short, eIF3 could block dissociation of mRNA from the 40S by DENR-MCT-1, while allowing DENR-MCT-1 to replace the P-site deacylated tRNA with Met-tRNA_i_^Met^ (Fig. 5). This could explain why this mechanism works most efficiently for the ‘start-stop uORFs’, as during termination the AUGs of these minimal uORFs are located in the in P-site and thus correctly positioned for recruitment of Met-tRNA_i_^Met^ for the next initiation event. Even if this speculative model holds true, it would not explain why DENR-MCT-1-stimulated REI operates specifically on uORFs with strong Kozak context. It is also not known whether DENR-MCT-1 travels with post-termination 40S ribosomes downstream to reinitiate at the main ORF, like eIF3 most probably does; however, if so, its partial overlap with 40S-binding sites of eIF1, and possibly eIF1A, could eliminate the need for eIF1/eIF1A in proper AUG selection at the main ORF. Taking into account that MCT1, DENR and eIF2D contain homologs in budding yeast (represented by the poorly characterized proteins Tma20, Tma22 and Tma64, respectively), it will be intriguing to explore whether these yeast counterparts also play some role in REI in this unicellular organism. Of note, in low boron conditions REI after ‘start-stop uORF’ is also critical for translation of the main ORF of *A. thaliana NIP5;1* mRNA encoding the boron transporter; however, whether or not DENR-MCT-1 contributes to this boron-regulated mechanism remains to be seen (Tanaka *et al.*^2016^).

## REI AFTER TRANSLATION OF LONG ORFs

In contrast to REI after short uORFs, REI after translation of long ORFs, i.e. those encoding cellular proteins, is presumably a very rare event because most ribosomes translating canonical ORFs are expected to undergo full ribosomal recycling upon completion of protein synthesis. In addition, it is presumably impossible to retain any initiation factors involved in the primary initiation event, including eIF3, during the extended period of elongation required to translate long uORFs. Nonetheless, there are quite a few exceptions to this rule, especially among viral mRNAs, where REI after long ORFs provides the means for (i) maximizing the genome coding capacity, (ii) regulating the levels of expressed proteins and (iii) redirecting the host translational machinery to the virus. A number of distinct strategies appear to be used involving REI mediated by either 40S or 80S post-TCs with peculiar means of REI start site selection and varying requirements for interactions between viral proteins or mRNA and ribosomal subunits or eIFs.

### Coupled termination-REI

Although termination-REI is represented in the literature mainly by the termination upstream ribosome binding site (TURBS)-mediated mechanism in caliciviral protein synthesis, described at length below, it comprises a fairly heterogeneous group of molecular processes utilizing distinct mRNA sequence motifs or structures upstream or downstream of the termination-REI start site to ensure efficient translational coupling between termination on the upstream ORF and subsequent REI on the downstream ORF. This is achieved by the retention of the post-termination 40S subunit on mRNA following the first recycling step (dissociation of the 60S subunit). Based on *in vitro* experiments, it is theoretically possible that in some cases ribosomal recycling does not occur at all and termination-REI is mediated by the post-termination 80S ribosomes; however, *in vivo* evidence is missing. The common feature linking all of these processes is the existence of a region between the two ORFs, where the stop codon of an upstream ORF is functionally connected with the start codon of the downstream ORF. Frequently, the stop and start codons are in close proximity, which is often expressed by the formula AUGn_x_UGA (with X being 2, 5, 8 or 14 nt); alternatively, they may overlap each other as UAAUG or AUGA. A longer separation, greater than 14 nt, has been observed in some cellular mRNAs; however, in these cases the REI start codon always precedes the termination codon of the uORF. The proximity of stop-start codons in these systems places the post-termination ribosomes in the vicinity of the next start codon and should obviate the need for traversing/scanning during REI. This presumably reflects the absence of all initiation factors associated with the post-TCs, including eIF3, owing to the extended period of elongation involved in translating the long uORF.

#### Termination-REI in caliciviruses

The most extensively studied viral REI events occur in the single-stranded positive sense RNA viruses of different genera of the family *Caliciviridae*, including rabbit hemorrhagic disease virus (RHDV) (Meyers 2003, 2007); feline calicivirus (FCV) (Luttermann and Meyers 2007; Pöyry *et al.*^2007^); and bovine, human and murine noroviruses (NVs) (McCormick *et al.*^2008^; Napthine *et al.*^2009^; Luttermann and Meyers 2014). Caliciviruses produce 3^΄^ co-terminal subgenomic mRNAs that are always dicistronic (for simplicity, these two cistrons will be designated here as ORF1 and ORF2, although according to their original position in full-length genomic mRNA they are usually described as ORF2 and ORF3, respectively). ORF1 encodes a major capsid protein and the downstream ORF2 encodes a small basic protein, which is essential for infectivity and was proposed to be involved in different regulatory functions. The ORF2/ORF1 expression ratio varies from ∼3% (in human NV) (Luttermann and Meyers 2014) to about 20% (in RHDV) (Meyers 2003, 2007). Depending on the viral species, the two ORFs overlap by 1–14 nt. Thus, even though the ORF1 stop codon is always close to the ORF2 AUG codon, there exists a certain degree of flexibility in their spacing; and as might be expected, the REI frequency drops with increasing distance between the stop and start sites. These observations are in sharp contrast with REI after short uORFs (such as in the case of *GCN4*), where the efficiency of REI increases with increasing distance between the two ORFs, thus demonstrating that distinct mechanisms apply.

**Figure 6.**
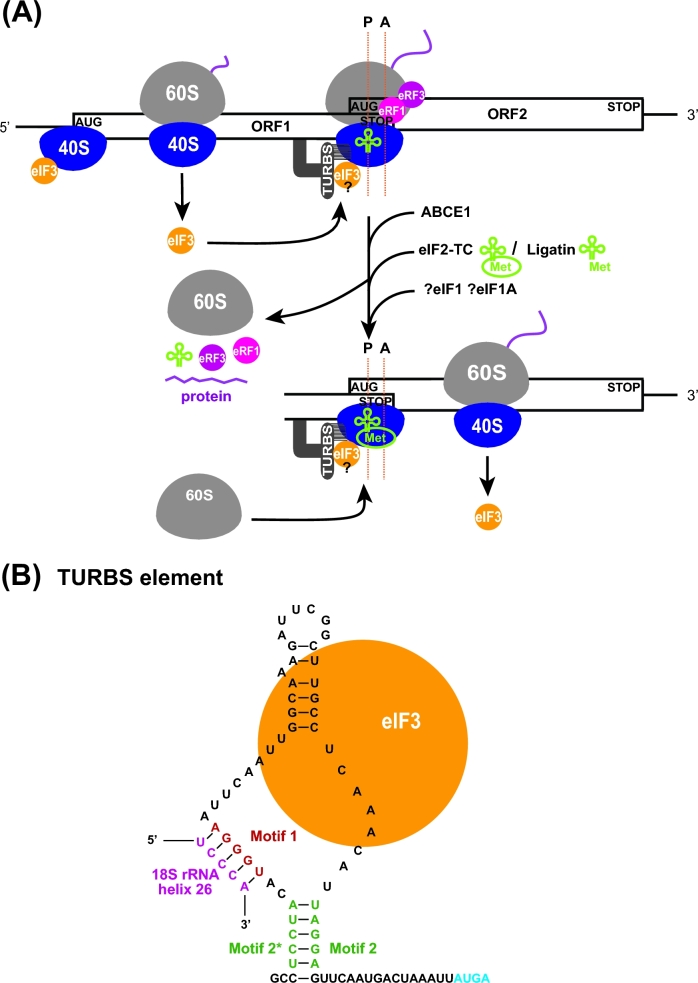
(**A**) Model for the termination-reinitiation mechanism in caliciviruses with overlapping ORFs mediated via TURBS base pairing with 18S rRNA, which can be perhaps further potentiated by eIF3 binding to both TURBS and the 40S. For details, see the main text. (**B**) Predicted secondary structure of the FCV TURBS illustrating base pairing between motif 1 and helix 26 of 18S rRNA, motifs 2 and 2^*^, as well as the schematic interaction of TURBS with eIF3 (based on Jackson, Hellen and Pestova 2012).

Detailed *in vitro* and *in vivo* mutational analyses have shown that the termination-REI process in caliciviruses depends on specific mRNA sequence motifs typically situated within 40–90 nucleotides upstream of the ORF1 termination codon, designated as TURBS (Fig. 6). Since ORF2 could be replaced by various reporter genes with no effect on the REI efficiency (Meyers 2003; Pöyry *et al.*^2007^), no dependence on particular downstream sequences is considered likely. However, a recent study suggested that, at least in the case of FCV, the efficiency of ORF2 expression is also modulated by the primary and secondary structures of the region downstream of the stop-restart signal (Habeta *et al.*^2014^). Additional analyses are thus required to resolve this potential discrepancy. Also, no requirement for a virus-encoded transactivator protein has been reported so far.

The TURBS region contains three essential motifs, designated as 1, 2 and 2^*^ (Fig. 6B). Motif 1 contains a conserved UGGGA core sequence located at a similar position relative to the stop-restart site within all caliciviral mRNAs. Importantly, the UGGGA sequence is complementary to the single-stranded loop at the tip of helix 26 of 18S rRNA, residing near the exit pore of the mRNA-binding channel (Luttermann and Meyers 2007; Meyers 2007) (Fig. 6B). With the help of yeast genetics, it was shown that this hybridization interaction is critical for tethering the post-termination 40S subunits to the viral mRNA (Fig. 6A); mutations within motif 1 reduced REI efficiency of a reporter mRNA in a manner rescued by complementary changes in 18S rRNA (Luttermann and Meyers 2009). In contrast to motif 1, motifs 2 and 2^*^ are species-specific and complementary to one another. Base pairing between these motifs allows formation of a stem loop structure in which the conserved UGGGA nucleotides of motif 1 are exposed in an internal loop. Occurrence of this structure in ORF1 at a defined distance upstream of the termination-REI site was proposed to enable placement of motif 1-bound 40S subunits directly at, or at least in proximity to, the REI start codon (Luttermann and Meyers 2007; Meyers 2007). Moreover, it was suggested that this tethering interaction, stabilizing post-TC 40S subunits on viral mRNA, might provide sufficient time for recruitment of initiation factors (mainly the eIF2-TC) that are critical for recognition of the REI start codon. The critical importance of TURBS sequences for REI was recently confirmed in a mammalian *in vitro* reconstituted system using model RHDV mRNAs with mutated motif 1 and motif 2^*^ (Zinoviev, Hellen and Pestova 2015).

Interestingly, in some caliciviruses, as well as in *Influenza B* (see below), two alternative structural isoforms of TURBS were proposed based on secondary-structure predictions and enzymatic and chemical probing (Powell *et al.*^2008^; Napthine *et al.*^2009^). In one of these structures motif 1 is sequestered by base pairing with another region of the viral mRNA, which makes it inaccessible for the tethering interaction, while the other structure shows the original arrangement with motif 1 accessible in the apical loop (Powell 2010). It was therefore suggested that translation through ORF1 might be required for structural remodeling of the TURBS to expose motif 1 for base pairing with 18S rRNA (Powell 2010). This suggestion invokes an interesting analogy with the folding of the 5´ enhancer structure preceding the *GCN4*’s uORFs 1 and 2 after its sequence has emerged from the mRNA exit channel, as discussed above (Munzarová *et al.*^2011^; Gunisova and Valasek 2014). The proposed need for the translation-dependent remodeling of TURBS could help explain why the REI event occurs only after termination at a nearby stop codon. It is possible that constitutive formation of the TURBS conformation that exposes motif 1 would allow it to act as an internal ribosome entry site (IRES), promoting internal initiation by direct recruitment of the small ribosomal subunit to the ORF2 start site, without prior uORF1 translation. Uncoupling ORF2 translation from ORF1 in this way would alter the relative amounts of the two gene products and could be detrimental to virus propagation.

Various studies have demonstrated that caliciviral termination-REI is extraordinarily insensitive to substitutions in the REI start codon, being largely insensitive to single substitutions and not fully impaired even when two or all three positions are mutated (Meyers 2003, 2007; Luttermann and Meyers 2007, 2014; Pöyry *et al.*^2007^; Napthine *et al.*^2009^). Regardless of the REI start codon alterations, the initiating amino-acid residue in most of the ORF2 FCV proteins is still methionine, indicating decoding of near-cognate or even non-cognate start codons by Met-tRNA_i_^Met^ (Luttermann and Meyers 2007). Since investigations of human NV revealed that the Kozak context has also no influence on the REI rates measured with an AUG start site (Luttermann and Meyers 2014), the mechanism of start codon selection is clearly noncanonical.

Interestingly, if a second AUG is introduced downstream of the WT ORF2 AUG codon, most REI occurs at the original REI start site (Luttermann and Meyers 2014). However, when the AUG triplet is introduced close to a mutated version of the original REI start site, the new AUG is now preferred over the mutated start site (Luttermann and Meyers 2014). This indicates that base pairing with the anticodon of Met-tRNA_i_^Met^ still contributes to selection of the REI start site even though a perfect codon:anticodon duplex is not critical for REI to occur. The relaxed requirement for a canonical AUG start codon on the one hand and the critical importance of maintaining a specific distance between the terminating ribosome and TURBS on the other clearly support the idea that the tethered post-termination 40S subunit is delivered directly to the ORF2 REI start site. The limited flexibility with respect to the juxtaposition of termination and REI start sites is believed to reflect a restricted mobility of the tethered 40S subunit following termination. The range of this spatially limited lateral migration during REI start codon selection is probably determined by the reach that the ribosomal P-site of the TURBS-bound 40S subunit has on each side of the ORF1 stop codon.

All data presented so far suggest that the caliciviral REI mechanism is mediated strictly by the post-termination 40S complexes. However, a recent study challenged this issue by investigating the plasticity in requirements for REI start codon selection by post-termination 40S subunits versus post-termination 80S ribosomes using mutant RHDV and NV mRNAs in an *in vitro* reconstituted system (Zinoviev, Hellen and Pestova 2015). At the AUG REI start site or when it was replaced by near-cognate codons, the post-termination 40S subunits reinitiated via base pairing with Met-tRNA_i_^Met^ (as observed before) and required the combined assistance of eIFs 1, 1A and eIF2, or eIF2D (Ligatin) only. By contrast, REI at non-cognate codons could be achieved solely with eIF2D (Ligatin) and cognate aminoacyl-tRNA (Zinoviev, Hellen and Pestova 2015). As observed before, the position of the original REI start site was strongly preferred over a second AUG inserted into ORF2, and more efficient REI took place from the inserted AUG only when the original AUG was mutated (Zinoviev, Hellen and Pestova 2015). Strikingly, these authors showed that REI on caliciviral mRNAs *in vitro* could be also executed by post-termination 80S ribosomes that efficiently reacquired Met-tRNA_i_^Met^ and moved a few nt upstream (for RHDV) or downstream (for NV) to reinitiate at AUG of ORF2 or, in the case of RHDV, at the near-cognate codon in place of AUG. Interestingly, post-termination 80S ribosomes could also migrate to, and initiate at, nearby non-cognate codons, in which case REI required binding of the respective cognate elongator tRNAs directly to the P-site (Zinoviev, Hellen and Pestova 2015). Whether such termination-REI events involving post-termination 80S ribosomes occur *in vivo* is presently not known.

Recent work of Luttermann and Meyers (2014) showed that when the original REI start codon is mutated, REI in human NV can also occur at more distant downstream sites (up to 78 codons), though with a much lower efficiency. Interestingly, this downstream REI was dependent on the Kozak sequence context. Movement of the 40S subunit along the mRNA in the 5^΄^ to 3^΄^ direction together with the requirement for a strong Kozak context is reminiscent of the scanning process in a standard cap-dependent translation initiation. Thus, it was suggested that in addition to the specific TURBS-dependent positioning of the post-termination 40S complex onto the REI start site, start site selection during termination-REI in caliciviruses can be achieved by an alternative, ‘back-up’ mechanism. When the TURBS-captured 40S subunits cannot establish stable codon-anticodon interaction at the original REI start site, a certain percentage of these 40S subunits may adopt a ‘short uORF-like’ mode of REI by acquiring initiation factors and scanning downstream to the next AUG codon with strong Kozak context (Luttermann and Meyers 2014).

Tethering the post-termination 40S subunit to the viral mRNA is not the only function that has been attributed to TURBS. UV cross-linking assays with the FCV TURBS demonstrated that it binds eIF3 (Fig. 6) (in particular, the eIF3a, eIF3b, eIF3d, eIF3l and eIF3g subunits were cross-linked to the FCV element), and TURBS mutants with reduced REI activity were shown to be defective in either eIF3 or 40S subunit binding, or both (Pöyry *et al.*^2007^). The proposed eIF3 involvement in termination-REI might resemble REI after short uORFs in yeast cells discussed above, where eIF3 critically stabilizes the post-termination 40S-mRNA complex (Munzarová *et al.*^2011^; Mohammad *et al.*^2017^). Even though efficient REI after short uORFs in mammalian systems also appears to require the eIF4F complex (Pöyry *et al.*^2004^), in addition to eIF3 (Hronova *et al.*^2017^), the TURBS-mediated REI event seems to have no requirement for any of the eIF4F components (Pöyry *et al.*^2007^). This makes sense because the TURBS-captured 40S subunits are most likely loaded directly onto the REI start codon of the downstream cistron, obviating the need for eIF4F function in promoting *de novo* mRNA recruitment and scanning through structured mRNA leaders.

Owing to the proposed role of eIF3 in ribosomal recycling (Pisarev, Hellen and Pestova 2007), it was originally suggested that its interaction with TURBS increases the rate of 60S subunit recycling and as such provides the tethered post-termination 40S subunit with more time to acquire initiation factors (like eIF2-TC) necessary for REI (Pöyry *et al.*^2007^). However, the eIF3-mediated 80S splitting occurs only in a narrow range of low Mg^2+^ concentrations (Pisarev, Hellen and Pestova 2007), and subsequent *in vitro* and *in vivo* experiments showed that subunit splitting is primarily performed by the canonical recycling factor ABCE1 (Pisarev *et al.*^2010^; Shoemaker and Green 2011; Young *et al.*^2015^), ostensibly at odds with the original suggestion. What then could be the eIF3 contribution, if any? It was shown that, after the ABCE1-mediated splitting, eIF3 prevents mRNA dissociation from the 40S post-TC complex (Kolupaeva *et al.*^2005^; Pisarev *et al.*^2010^), in accordance with the importance of eIF3 in mRNA recruitment and stabilization on the 40S subunit (Kolupaeva *et al.*^2005^; Jivotovskaya *et al.*^2006^; Khoshnevis *et al.*^2014^; Aitken *et al.*^2016^). This mRNA-40S stabilization function, perhaps together with the role of eIF3 in 60S subunit recycling and in preventing ribosomal subunit re-association (Kolupaeva *et al.*^2005^), might explain how eIF3 promotes termination-REI after long ORFs (see our model below).

New insights into the role of eIF3 and other eIFs in this mechanism were recently provided by the *in vitro* reconstitution experiments mentioned above using two model caliciviral mRNAs containing RHDV or human NV TURBS elements (Zinoviev, Hellen and Pestova 2015). Unexpectedly, the experiments that monitored the fate of 40S subunits following the subunit splitting in the presence or absence of ABCE1 suggested that eIF3 was not essential for efficient REI, and that only eIF1, 1A and eIF2-TC, or just eIF2D (Ligatin) alone, sufficed. In fact, the role of eIF2D (Ligatin) or DENR-MCT-1 in REI on RHDV mRNA is consistent with their abilities to stimulate eIF2-independent recruitment of Met-tRNA_i_^Met^ to mRNA-40S complexes in which the start codon is placed directly in the P-site (Skabkin *et al.*^2010^), which is ensured by the TURBS-18S rRNA interaction. The fact that this model system revealed no eIF3 dependence could be explained by non-physiological conditions of the *in vitro* system or by proposing that the eIF3 contribution to termination-REI varies for different caliciviruses, perhaps in inverse relation to the strength of their TURBS-40S interactions. Interestingly, some stimulatory role for eIF3 even in this system was observed when concentrations of other factors became limiting, e.g. when eIF1 or eIF1A was present individually, when eIF2-TC was added following a delay or when the TURBS elements were mutated; in the latter, eIF3 became nearly essential for REI (Zinoviev, Hellen and Pestova 2015). These findings may suggest the following. Besides ribosomal recycling, eIF3 was recently shown to control translation termination and thus it is expected to associate with early terminating ribosomes, perhaps in a complex with eRFs that was shown to exist *in vivo* (Beznosková *et al.*^2013^). This might mean that termination complexes in living cells come into contact with eIF3 much earlier than other eIFs, shown to be relatively more important for REI in the *in vitro* system. This could impose a marked *in vivo* requirement for the mRNA stabilization role of eIF3 during the onset of the termination-REI mechanism (fortifying the TURBS-40S contact), as discussed above.

To summarize, the TURBS-40S interaction represents the critical requirement for the termination- REI mechanism to occur (Fig. 6A). eIF3 may further stabilize the mRNA-40S complex and, in the case that eIF2D (Ligatin) ensures the subsequent replacement of deacylated tRNA with Met-tRNA_i_^Met^, it may also prevent eIF2D’s ability to dissociate 40S post-TCs, as suggested above. Alternatively, dissociation of the deacylated tRNA would be mediated by eIFs 1 and 1A, and eIF2 would subsequently deliver Met-tRNA_i_^Met^ to the P-site to form the TURBS REI complex poised for elongation. It will be of great importance to investigate the functions of eIF1, eIF1A, eIF2-TC, eIF3 and eIF2D (Ligatin) in TURBS-mediated REI *in vivo* to determine their physiological contributions to this mechanism. Another interesting question is how the caliciviral TURBS-mediated termination-REI mechanism would respond to decreased levels of the eIF2-TC provoked by stress-activated eIF2α phosphorylation by kinases such as PKR and PERK, whose activation is triggered by viral infections to produce a systematic shutdown of protein synthesis. To the best of our knowledge, there is currently no information about the control of eIF2α phosphorylation during calicivirus infection.

#### Termination-REI in Influenza B

The termination-REI mechanism has also been quite extensively studied in the single-stranded negative sense RNA Orthomyxovirus *Influenza B*, where numerous similarities to the caliciviral mechanism have been observed (Horvath, Williams and Lamb 1990; Powell *et al.*^2008^; Hatta *et al.*^2009^). Its segment 7 encodes two proteins whose coding sequences overlap in a typical UAAUG stop-restart arrangement and ∼10% of ribosomes terminating at the ORF1 stop codon were shown to reinitiate at the ORF2 AUG (Powell *et al.*^2008^). Efficient REI on ORF2 is dependent on proximity of the stop-restart sequence and the ∼45 nt long TURBS region upstream of the overlap, which contains the motif 1 UGGGA core sequence, as well as complementary motifs 2 and 2^*^ (Horvath, Williams and Lamb 1990; Powell *et al.*^2008^; Hatta *et al.*^2009^). Oligonucleotide targeting experiments and expression studies in yeast cells support the hypothesis that motif 1, like in caliciviruses, interacts directly with helix 26 of 18S rRNA (Powell *et al.*^2011^). Whether or not there is also a need for the TURBS-specific mRNA secondary structure remains to be resolved since the most recent experimental data provided only limited support for the initial secondary structure predictions and translational remodeling hypothesis (Powell *et al.*^2008^; Powell 2010; Powell *et al.*^2011^). In further analogy, a variety of non-canonical initiation codons can be utilized and there is also a minimal requirement for optimal start codon context (Powell *et al.*^2008^). Accordingly, it was suggested that the decreased stringency in REI start codon selection may reflect the reduced requirement for the full complement of initiation factors in the termination-REI process, such as eIF1 and eIF1A—key players in canonical start site recognition (Hinnebusch 2017). As stated above, the pace of the termination-REI mechanism *in vivo* may simply be too quick for these factors to bind the post-termination mRNA-40S complex on time. Alternatively or in addition, the reduced stringency might result from the fact that the 40S subunit is tethered rather than scanning, which increases the dwell time over the start codon. This option is in analogy with earlier findings of Kozak that a stable stem loop inserted downstream of the start site at a position that would arrest the scanning PIC with the start codon in the P-site increases initiation at AUGs in poor context (Kozak 1991).

Also as in case of caliciviruses, it was shown that when termination occurs at the normal distance relative to the TURBS, a certain proportion of ribosomes is able to locate AUG codons placed at a relatively remote location (63 nt) downstream of the mutated original REI start codon (Powell *et al.*^2011^). Since REI on distant AUGs was not inhibited in the eIF4G-depleted rabbit reticulocyte lysates, it was suggested that the tethered 40S subunit can move some distance in a linear, eIF4F-independent manner akin to scanning, or that a direct transfer of the 40S subunit from the TURBS to the distant AUGs might be facilitated by looping out of the mRNA segment between the tethered 40S subunit and downstream AUG (Powell *et al.*^2011^).

*Influenza B* REI can be also stimulated by exogenously added eIF3, even when TURBS was rendered defective (by mutated motif 1), further supporting the view that eIF3 can substitute for the TURBS-18S rRNA interaction to prevent dissociation of post-termination 40S subunit from viral mRNA and ensure its transfer to the REI start site (Powell *et al.*^2011^). Thus, all of these data suggest that the *Influenza B* termination-REI mechanism is mechanistically very similar to that of caliciviruses and depends on direct placement of the post-termination 40S subunit onto or nearby the REI start codon.

#### Termination-REI in other viruses and retrotransposons

There is evidence that other unrelated viral RNAs employ coupled termination-REI with similar molecular mechanisms. In pneumo- and metapneumoviruses, termination-REI was demonstrated to regulate the synthesis of a downstream cistron from a single transcript (Ahmadian, Randhawa and Easton 2000; Gould and Easton 2005, 2007). As in caliciviruses, the REI start site must be in close proximity to the termination codon of the uORF, although in some cases larger separations of up to 29 nt can be tolerated (Ahmadian, Randhawa and Easton 2000). Efficient REI is also dependent on sequences upstream of the termination codon, but the identified region is quite large (up to ∼200 nt) and apparently contains extensive secondary structures (Gould and Easton 2005). Unlike in caliciviruses, however, this region lacks an obvious motif 1 and thus its exact function is unknown. Similarly, in victoriviruses the stop and REI start sites of REI-regulated cistrons often overlap, and the upstream sequence—in particular the 32 nt long region immediately upstream of the stop-start site—was shown to be important for efficient REI (Li *et al.*^2011^). This region is predicted to adopt a specific pseudoknot structure but also lacks the motif 1 sequence. In hypoviruses, where the downstream cistron appears to be translated by the termination-REI mechanism via an overlapping stop-start site at a frequency of <5%, no motif 1 has been identified; however, an upstream sequence involved in the putative REI shows some complementarity to 18S rRNA (Guo *et al.*^2009^). Interestingly, the protein encoded by the upstream hypoviral cistron seems to participate in REI of the downstream ORF by a yet to be identified mechanism (Guo *et al.*^2009^).

A specific translational coupling of a similar nature to those described above has also been observed in the non-LTR silkworm retrotransposon SART1, with one apparent exception; the complex secondary structure needed for proper functioning of this mechanism was located downstream—not upstream—of the overlapping stop-restart site (Kojima *et al.*^2005^). An unconventional termination-REI mechanism has also been proposed for another non-LTR retrotransposon, mammalian LINE-1, where sequences within ORF2 may play a role in positioning the ribosome onto or nearby the ORF2 AUG codon (Alisch *et al.*^2006^). Here, however, ORF1 and ORF2 are separated by a 63-nt long inter-ORF spacer and in order to reach the downstream cistron, some sort of scanning through this spacer might have to occur, unless the ORF1 stop and ORF2 start are brought together by a looping mechanism mentioned above.

#### Termination-REI in cellular mRNAs

The fact that the termination-REI mechanism is widespread in viruses and does not seem to require specific viral-encoded proteins raises the question of whether some of the rarely occurring cellular bi- or polycistronic mRNAs (with at least two long ORFs) utilize a similar mechanism. Recently, Gould *et al.* (2014) identified more than 2000 genes in the human genome whose transcripts contained a major ORF overlapping with a second ORF comprising at least 50 codons. From 24 experimentally investigated transcripts, 22 expressed an ORF2-encoded protein, which may indicate that the protein-coding potential of 3^΄^ UTRs of cellular mRNAs is greater than generally believed. Importantly, five transcripts were shown to use a coupled termination-REI mechanism for access to the ORF2 AUG codon; however, none of these mRNAs contained an upstream sequence that resembles the TURBS or any other viral REI-promoting sequences. In fact, detailed analysis of one of these transcripts, encoding the CASQ2 protein, revealed that the termination-REI mechanism did not depend on any mRNA sequences at all, and required instead an aspartate-rich repeat region at the C-terminus of CASQ2. Quantification of the coupling efficiency demonstrated that the levels of downstream protein were ∼82-fold lower compared to the upstream product. It was suggested that the aspartate-rich region might act to slow down the elongating ribosomes or cause them to pause at the end of ORF1, but how this would promote REI at ORF2 remains to be determined.

Another example of a cellular mRNA likely to be regulated by translational coupling is the mouse embryonic mRNA splicing variant of glutamic acid decarboxylase (GAD) (Kojima *et al.*^2005^). The bicistronic RNA of GAD contains UGAUG overlapping stop-start codons and its downstream ORF was shown to be translated *in vivo* (Szabo, Katarova and Greenspan 1994). Strikingly, a putative TURBS motif 1 has been identified within the sequence preceding the stop-restart signal, as were the complementary motifs 2 and 2^*^ (Powell 2010). A sequence related to the TURBS core sequence UGGGA has also been found in the 5^΄^ leader sequence of the long isoform of the human *SLAMF1* gene (encoding the CD150 membrane protein), where the stop codon of the last 29-codon uORF (of altogether four uORFs) overlaps the start codon of the main reading frame by 1 nt (UGAUG) (Putlyaeva *et al.*^2014^). Although this uORF, according to its relatively short length, might still qualify as an REI-permissive uORF, the fact that it overlaps the main ORF by even 1 nt likely precludes it from functioning as a typical REI-permissive uORF, in which an appreciable distance/time is required for the traversing/scanning 40S subunit to rebind eIF2-TC *en route* to the downstream ORF. It remains to be determined whether the TURBS-mediated termination-REI mechanism operates on the *SLAMF1* gene. If so, the short length of the uORF might allow eIF3 to persist on elongating ribosomes over its entire length (see above) and cooperate with TURBS immediately on the onset of termination to promote efficient termination-REI. This example illuminates a theoretical possibility of how some features of the fundamentally different REI mechanisms may act together to produce the final outcome.

Clearly, dissecting the intricate ways in which viral genomes exploit the host molecular machineries to produce all of their proteins in the proper stoichiometries has revealed an important mechanism of REI. It will be valuable to determine whether this coupled termination-REI mechanism is widespread in cellular mRNAs, where it could provide an unappreciated means of expanding the coding capacity of mammalian genomes.

### Transactivation of translation on long ORFs by a viral factor

**Figure 7.**
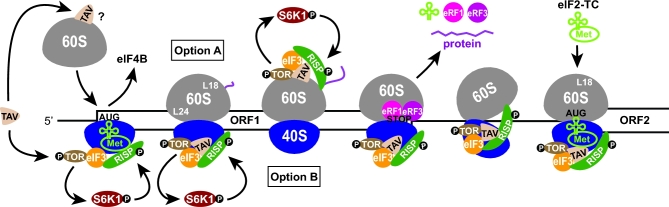
Model for translational control of the CaMV mRNA mediated via reinitiation promoted by the virus-encoded transactivator protein called TAV and the plant-specific REI supporting protein (RISP). For details, see the main text.

In plants, an exceptional strategy to ensure translation of all cistrons in polycistronic mRNAs has been uncovered in CaMV and some related pararetroviruses of the icosahedral *Caulimoviridae* family, wherein the REI mechanism is strictly dependent on a virus-encoded transactivator protein called TAV (transactivator/viroplasmin) (Fig. 7) (Bonneville *et al.*^1989^; Park *et al.*^2001^). Upon infection, the CaMV genome is transcribed into pregenomic RNA containing seven long ORFs, which can be further internally spliced into individual derivatives containing at least four long ORFs. All ORFs (except for the TAV ORF) are tightly spaced and usually exhibit short regions of overlap, compatible with linked translation. Interestingly, increasing overlap between the two long CaMV ORFs (by more than ∼130 nt) was found to inhibit transactivation by TAV (Futterer and Hohn 1991), which may imply that the backward movement of reinitiating ribosomes is somehow limited. In addition to activating translation of consecutive long viral ORFs, TAV is able to promote REI on completely artificial bicistronic reporters, indicating that specific *cis*-sequence signals are not required for transactivation of the second ORF translation (Bonneville *et al.*^1989^; Futterer and Hohn 1991, 1992). TAV-mediated REI is also not affected by the distance between the two consecutive long ORFs—occurring efficiently with distances between the uORF and downstream ORF ranging from only a few nt up to 700 nt (Futterer and Hohn 1991). This contrasts with the REI mechanism mediated by short uORFs, where a minimum intercistronic distance is required to provide sufficient time for efficient eIF2-TC recruitment as the 40S ribosome traverses from the uORF to the downstream ORF.

Studies from Ryabova's laboratory on the molecular mechanism of CaMV REI indicate that a key feature is the retention of REI-supporting factors, namely eIF3 (Park *et al.*^2001^), RISP (Thiébeauld *et al.*^2009^) and TOR (Schepetilnikov *et al.*^2011^), on ribosomes translating the first long ORF (Fig. 7). TAV was shown to interact with these factors and other components of the host translational machinery (namely 60S ribosomal proteins Rpl13/eL13, Rpl18/eL18 and Rpl24/eL24) directly via its two central domains that are essential for REI.

The initial *in vitro* studies demonstrated that TAV can form two stable complexes: in the first, TAV bridges interaction between eIF3 (via its g subunit) and the 60S subunit (via Rpl18/eL18 or potentially also Rpl13/eL13); and in the second complex, eIF3 connects TAV with the 40S subunit (Park *et al.*^2001^). Later it was shown that the TAV-eIF3-40S complex can join with the 60S subunit to form an 80S-eIF3-TAV complex *in vitro*, which was also observed after fractionation of cell extracts by sucrose gradient centrifugation (Park *et al.*^2001^). Consistently, analysis of extracts from CaMV-infected plants revealed significant accumulation of TAV and eIF3 in the polysomal fractions (Park *et al.*^2001^). These observations suggested that the TAV-eIF3 complex is associated with elongating ribosomes *in vivo* and that TAV might prevent spontaneous eIF3 dissociation from ribosomes translating long ORFs, through its mutual interaction with eIF3 and 60S proteins Rpl18/eL18 or Rpl13/eL13, to maintain their REI competence post-termination (Fig. 7). Because Rpl24/eL24 is located at the intersubunit surface and serves as one of the intersubunit bridges, its role in eIF3 retention on elongating ribosomes is highly unlikely. Instead, it was proposed that its interaction with TAV might actually lead to inhibition of protein synthesis during the late phase of viral infection, promoting a switch to viral assembly (Park *et al.*^2001^).

Later it was shown that eIF4B (a factor stimulating the RNA helicase activity of eIF4A that is, however, not essential in plants (Altmann *et al.*^1995^)) shares the same binding site on eIF3g with TAV and potently outcompetes TAV for binding to the eIF3-40S complexes both *in vitro* and *in vivo* (Park *et al.*^2004^). Consistently, transient overexpression of eIF4B in plant protoplasts specifically inhibited TAV-mediated REI at a second ORF (Park *et al.*^2004^). eIF4B did not displace eIF3 from the eIF3-TAV-60S complexes; however, 60S subunit joining disrupted only the eIF4B-eIF3-40S and not the TAV-eIF3-40S complexes *in vitro* (Park *et al.*^2004^). These data indicated that eIF4B precludes the TAV-eIF3-40S complex formation during the first initiation event and that TAV enters the host translation machinery after eIF4B removal from the 48S PIC (i.e. at or shortly before subunit joining) to stabilize eIF3 on the translating ribosome (Fig. 7). If true, it would explain why TAV affects exclusively the second and all following rounds of initiation on polycistronic mRNA.

Subsequently, a novel plant-specific REI supporting protein (RISP) was identified and proposed to act as an indispensable host factor enhancing TAV function in REI (Thiebeauld *et al.*^2009^). Knockout of *rispa* (encoding RISP) caused a delay in viral replication and reduced TAV-mediated transactivation of REI (Thiebeauld *et al.*^2009^); the innate RISP function in non-infected plant cells is not known. RISP has a predicted coiled-coil structure characterized by four helices: H1–H4. The interaction between TAV and RISP was mapped within putative helix H3, while segments represented by helices H2 and H4 were found to interact with eIF3a and eIF3c subunits, and with the C-terminus of Rpl24/eL24, respectively (Thiebeauld *et al.*^2009^). Binding of RISP to the 40S subunit occurred only with preformed RISP-eIF3 complexes suggesting that RISP enters the cell translational machinery together with eIF3 at the stage of 43S PIC formation (Fig. 7) (Thiebeauld *et al.*^2009^). Importantly, RISP accumulation in polysomes required TAV indicating that (i) RISP is recruited to or stabilized in polysomes in CaMV-infected cells, and (ii) that, analogous to eIF3, TAV prevents RISP dissociation from the translating ribosomes during prolonged elongation (Thiebeauld *et al.*^2009^). Quaternary complexes composed of eIF3-TAV-RISP-eL24 could form *in vitro* (Thiebeauld *et al.*^2009^) which may suggest that, upon termination, RISP and TAV could bridge the interaction between eIF3-bound 40S and Rpl24/eL24 of 60S to enhance recruitment of 60S subunits, while the post-termination 40S subunit is engaged in AUG-searching process before each initiation event on polycistronic mRNA (Fig. 7).

Besides eIF3 and RISP, TAV function requires its physical interaction with the plant protein kinase TOR and its downstream effector S6K1; the TAV-TOR association is critical for viral fitness and, consistently, TOR-deficient plants are resistant to viral infection (Schepetilnikov *et al.*^2011^). As demonstrated both *in vitro* and *in vivo*, TAV overexpression (or viral infection) triggers TOR hyperactivation and high levels of S6K1 phosphorylation (Schepetilnikov *et al.*^2011^). Sucrose gradient fractionations revealed that activated TOR binds to polysomes simultaneously with TAV, eIF3 and RISP although TOR binding does not seem to be a prerequisite for polysome association of these other three factors (Schepetilnikov *et al.*^2011^). Importantly, however, polysomal association of TOR correlated with increased phosphorylation of polysome-associated RISP, a putative novel substrate of S6K1; RISP was found to stimulate TAV-mediated REI only in its phosphorylated state (Schepetilnikov *et al.*^2011^). Collectively, these findings may suggest that the essential function of TOR in TAV-mediated REI is to keep polysomal RISP in its phosphorylated state (Fig. 7). Interestingly, a non-phosphorylatable mutant of RISP showed increased affinity for eIF3 but decreased affinity for TAV and Rpl24/eL24 (Schepetilnikov *et al.*^2011^) suggesting that selective alterations in RISP-binding affinities upon its phosphorylation might be an important part of the regulatory mechanism. Notably, as discussed above, TOR-S6K1 connections with eIF3 were implicated in REI on the ARF-encoding mRNAs in *Arabidopis* (Schepetilnikov *et al.*^2013^), as well as in the PIC assembly during canonical translation initiation in mammals (Holz *et al.*^2005^).

All these findings were combined in the following elaborate model for TAV function in REI (Fig. 7). Upon infection, the overexpressed TAV binds to and activates TOR. Attendant S6K1 activation by TOR leads to RISP phosphorylation in the context of eIF3-bound PICs. During the 60S subunit joining step, eIF4B is ejected from the 40S subunit, enabling TAV to bind and thus stabilize the eIF3 and phosphorylated RISP binding to the 80S ribosome. Hypothetically, TAV may enter the assembly pre-bound to the 60S subunit (through Rpl13/eL13 and/or Rpl18/eL18) during subunit joining at the start codon of ORF1 or (since eIF3 can persist transiently on elongating ribosomes (Mohammad *et al.*^2017^)) during the first few rounds of elongation. The major role of TAV at this stage is to prevent dissociation of eIF3 and RISP during elongation on ORF1. Since binding of eIF3 to the solvent-exposed side of the 40S subunit has been firmly established (reviewed in Valasek *et al.*^2017^) (Fig. S2, Supporting Information), it seems reasonable to propose that the TAV-eIF3-RISP complex resides at least initially at this canonical eIF3-binding site on the 40S subunit. However, the model posits that TAV-bound eIF3 and RISP disengage from the 40S at the onset of elongation and relocates to the solvent surface of the 60S subunit via TAV binding to Rpl18/eL18 (and/or Rpl13/eL13), presumably to allow retention of the eIF3-TAV-RISP assembly throughout the prolonged period of elongation required to translate ORF1. It would be intriguing to see how TAV is arranged on the surface of the 80S ribosome, contacting Rpl18/eL18 or Rpl13/eL13 and interacting with eIF3 and RISP, without hampering the elongation process. The phosphorylated (or rephosphorylated) state of RISP is maintained by activated TOR-S6K1. Upon termination, the eIF3-TAV-RISP complex would need to be transferred back to the 40S subunit where its components can facilitate reassembly of a complex capable of recruiting eIF2-TC for REI. As mentioned above, the RISP-TAV complex is believed to bridge the 40S-60S interaction through contacts with eIF3 and Rpl24/eL24 and thus prevent ribosomal recycling at the ORF1 stop codon and enable reuse of the same 60S subunit during subsequent REI events. It was also speculated that the 40S-60S association would need to be sufficiently relaxed to allow efficient eIF2-TC binding during traversing of the putative 80S complex along viral mRNA. Considering the multiple interactions of TAV and other involved factors, as well as the rather dramatic relocation of eIF3-TAV-RISP from the 40S to 60S subunit and back postulated in this model, which were identified and proposed primarily based on biochemical experiments, it will be important to analyze the individual proposed steps in reconstituted, as well as *in vivo* systems. Structural analysis of different TAV complexes free or ribosome-bound would also shed more light on this intriguing REI mechanism and its unique reliance on non-canonical REI factors.

### REI in the 3^΄^ UTR

REI in 3^΄^ untranslated regions (UTRs) has generally been considered unfeasible until recently, when the application of ribosome profiling techniques revealed ribosome protected fragments (RPFs) in many non-coding regions including 3^΄^ UTRs of conventional mRNAs (Guydosh and Green 2014; Ji *et al.*^2015^; Miettinen and Bjorklund 2015; Young *et al.*^2015^; Mills *et al.*^2016^). Depending on experimental conditions, the proportion of reads arising from 3^΄^ UTRs varied between very low (<1%) and up to 20%–25% of all generated RPFs. Interestingly, an increased density of 3^΄^ UTR RPFs was found in some specific cellular lineages or for example in yeast cells lacking Dom34, a factor that rescues ribosomes stalled during elongation on cellular mRNAs (Guydosh and Green 2014; Miettinen and Bjorklund 2015; Mills *et al.*^2016^). The vast majority of these RPFs were, however, suggested to arise from non-translating 80S ribosomes. In contrast, one study estimated that ∼4% of all translated coding sequences in normal human cells derive from the 3^΄^ UTRs of transcripts encoding primary annotated ORFs (Ji *et al.*^2015^).

**Figure 8.**
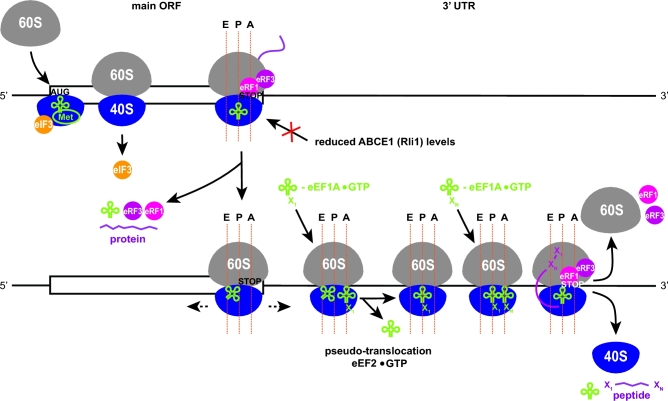
Model for translation reinitiation in the 3´ UTR. For details, see the main text.

In the recent work of Young *et al.* (2015), a special case of non-canonical REI involving 80S ribosomes was found to occur broadly in 3^΄^ UTRs in mutant yeast cells where the first stage of ribosome recycling (removal of 60S subunit) following termination was impaired (Fig. 8). Conditional depletion of the essential recycling factor Rli1 (ABCE1) led to increased accumulation of 80S ribosomes at stop codons and in the adjacent 3^΄^ UTRs of virtually all yeast genes. Three critical observations provided evidence that the 3^΄^ UTR ribosomes are frequently engaged in translation and that this form of translation represents new REI events as opposed to readthrough of the main ORF stop codon. First, numerous ribosome occupancy peaks were identified to coincide with stop codons in the 3^΄^ UTRs in all three reading frames relative to the main ORF, as expected from defective 60S subunit recycling on the main ORF and subsequent random REI. Second, induction of histidine starvation specifically increased ribosome occupancy at histidine codons located in the same 3^΄^ UTR reading frames terminated by stop codons exhibiting prominent 80S peaks in non-starved cells, consistent with stalled elongation at 3^΄^ UTR histidine codons. Finally, synthesis of small peptide products predicted from the 3^΄^ UTR ribosome occupancies at different genes, and representing all three reading frames, was identified by western blotting and mass spectrometry analyses.

Surprisingly, REI frequently occurred relatively close to the main ORF stop codon (within ∼10 nt upstream or downstream), but neither AUG codons nor triplets complementary to the ultimate P-site tRNA were preferred as start sites (Young *et al.*^2015^). This strongly suggests that (i) the 3^΄^ UTR REI does not involve scanning and start site selection via complementarity to either Met-tRNA_i_^Met^ in eIF2-TC or the deacylated tRNA in the P-site of post-termination 80S ribosomes, and (ii) that this mechanism radically differs from that described in an *in vitro* reconstituted system, where it appeared that REI by unrecycled post-termination 80S ribosomes was directed by complementarity to the ultimate deacylated tRNA in the P-site (Skabkin *et al.*^2013^). Could this difference arise from the fact that the *in vivo* study followed the fate of post-termination ribosomes after translation of long ORFs, whereas the *in vitro* study monitored the situation after translation of short uORFs? What is the mechanism of REI in 3^΄^ UTRs by unrecycled 80S ribosomes *in vivo*?

Young *et al.* (2015) have proposed that, following termination at the main ORF stop codon and polypeptide release, the splitting of the 60S subunit from the 80S post-TCs fails due to insufficient Rli1 (Fig. 8). The 80S post-TC thus releases only eRF1 from the A-site, allowing the deacylated P-site tRNA to adopt the pre-translocation P/E hybrid state required for freeing the 80S post-TC to migrate in either direction toward new sense codons. Choice of the REI start site is probably stochastic, determined by base pairing between a particular sense codon that appears in the A-site and the cognate aa-tRNA-eEF1A-GTP ternary complex (as stated above, no scanning is involved). Upon base pairing, a pseudo-translocation event—similar to that occurring during the A-site-initiated translation initiation directed by the dicistrovirus IGR IRES (Thompson 2012)—would occur to translocate the codon:aa-tRNA-ternary complex assembly into the P-site, exposing the next triplet in the A-site and allowing conventional elongation to commence (Fig. 8) (Young *et al.*^2015^). Whether this idea is correct and, if so, what other factors might be involved remains to be shown.

It is interesting to note that whereas depletion of Rli1 produced translating 80S ribosomes in 3^΄^ UTRs (Young *et al.*^2015^), it appeared that deletion of *DOM34* led primarily to accumulation of 80S ribosomes at the 3^΄^ UTR junction with the mRNA poly(A) tail (Guydosh and Green 2014). The fact that this phenomenon in *dom34Δ* cells was suppressed by Rli1 overexpression indicates that the 80S ribosomes accumulating at the poly(A) tail originate from failed 60S recycling at main ORF stop codons, despite WT levels of Rli1. To explain why a deficiency in Rli1 recycling leads primarily to 80S accumulation at the poly(A) tail in *dom34Δ* cells but not in the *DOM34* cells depleted of Rli1, it was suggested that Dom34 serves to rescue the class of 80S ribosomes that arrive at the poly(A) junction after failing to reinitiate at all or while still engaged in translation. By contrast, the reinitiating ribosomes observed on Rli1 depletion in *DOM34* cells represent the class that escaped Dom34 rescue.

This brings us to three fundamental questions: Does this type of 80S REI occur in normal cells? What is its frequency? And what purpose is served (if any) by the 3^΄^ UTR-encoded peptides produced in this way? Some of these peptides could easily be toxic and their production should be suppressed. Since REI products encoded in the *CWP2* 3^΄^ UTR were detected at low levels in WT yeast cells, and their synthesis was diminished by overexpressing Rli1 (Young *et al.*^2015^), it was suggested that this mechanism does operate at some level in normal cells. Since elevated levels of 3^΄^ UTR-localized ribosomes were also detected during stress (Young *et al.*^2015^), it was suggested that the WT level of Rli1 activity is just below the threshold of sufficiency for complete recycling, and that under stress this threshold may increase (or Rli1 levels might decrease) to enhance the frequency of 80S REI in 3^΄^ UTRs.

### REI of translation in prokaryotes

**Figure 9.**
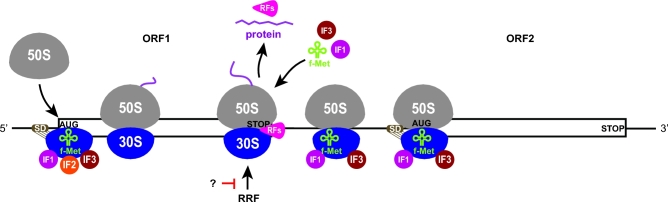
Model for translation reinitiation in prokaryotes; SD—Shine-Dalgarno sequence; RRF—ribosome recycling factor. For details, see the main text.

In contrast to eukaryotes, REI in prokaryotes is considered to be a frequent event, which is not surprising owing to the fact that most prokaryotic transcripts are naturally bi- or polycistronic. In addition, more than 75% of the intercistronic distances in *Escherichia coli* transcripts are shorter than 30 nt, which is considered too short to allow *de novo* initiation on the downstream cistron in view of the length of mRNA covered by one ribosome (34–38 nt) (Yamamoto *et al.*^2016^). However, our knowledge of the mechanism of prokaryotic REI—whether it is mediated by 30S and/or 70S ribosomes—has been quite limited. The recent *in vitro* study of Yamamoto *et al.* (2016) revealed somewhat unexpectedly that traversing to, and translation of, the downstream cistron is achieved by 70S ribosomes. In fact, 70S ribosomes were also able to initiate translation on monocistronic mRNA provided that its 5^΄^ UTR did not contain strong secondary structures. Using various reporters in an *in vitro* reconstituted system, these authors demonstrated that after termination, 70S ribosomes scan the sequence surrounding the termination codon for the presence of the SD motif which, through base pairing with the 3^΄^ end of 16S rRNA, positions the start codon of the downstream cistron in the ribosome P-site to produce a 70S initiation complex ready to commence translation (Fig. 9). A strong dependence on the presence of the SD motif upstream of the second cistron for its efficient translation was also previously demonstrated in an *in vitro* dual reporter system (Osterman *et al.*^2013^). Yamamoto *et al.* (2016) further showed that the inferred scanning process was triggered by fMet-tRNA_f_^Met^, did not require energy and was found to be regulated by IFs 1 and 3. Whereas the function of IF3 was essential to keep 70S ribosomes in the scanning-competent mode, IF1 stimulated 70S-mediated REI most probably by preventing deleterious entry into the A-site of the elongation ternary complexes (EF-Tu-GTP-aa-tRNA), which could interrupt the scanning process before the SD was located. The scanning-like movement of post-termination ribosomes was also detected in earlier *in vivo* studies (without distinguishing between 30S and 70S REI), where its radius of action exceeded more than 40 nt in both directions and REI occurred at the first start codon encountered that was preceded by an SD-like sequence (Adhin and van Duin 1990). The just described mechanism of 70S REI bears some features that are similar to eukaryotic REI by post-termination 80S ribosomes as described above (Skabkin *et al.*^2013^; Young *et al.*^2015^; Zinoviev, Hellen and Pestova 2015). In addition, the eukaryotic functional counterparts of IF3 and IF1, namely eIF1 and eIF1A, have also been implicated in scanning by 43S PICs during conventional initiation (Hinnebusch 2017). However, it is questionable whether eIF1 and eIF1A binding to the interface surface of the 40S subunit could be maintained in an 80S ribosome. Perhaps an even more crucial difference between the two systems is the absence of the SD:rRNA interaction in eukaryotes and a different mode of termination and ribosomal recycling mediated by unrelated factors (Myasnikov *et al.*^2009^; Buskirk and Green 2017; Hinnebusch 2017).

## REI OCCURING WITHIN CODING REGIONS

The following section deals with two mechanistically distinct processes that can be considered as specific types of REI even though they drive new translation from within the same coding sequence. The first, called retroreinitiation (retroREI), resembles 80S-mediated REI after long ORFs in requiring recognition of a stop codon. In this case, however, the stop codon is introduced by a nonsense mutation that prematurely terminates the decoding of the ORF. The second mechanism, designated as Stop-Carry On or StopGo, completely interrupts elongation and produces two discrete, sequentially synthetized peptides from the same ORF.

### Retroreinitiation

**Figure 10.**
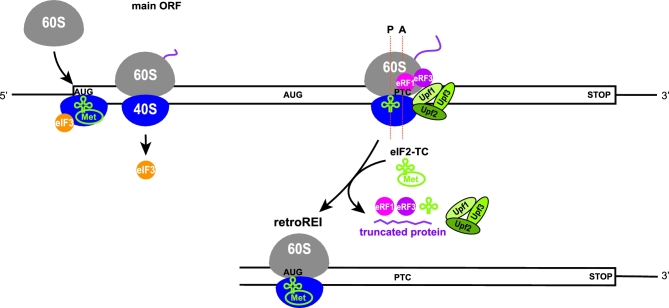
Model for retroreinitiation within the coding region; PTC—premature termination codon. For details, see the main text.

RetroREI is believed to represent a specific REI mechanism occurring within a conventional ORF that happens to be interrupted by a premature termination codon (PTC). Such PTCs are normally recognized as aberrant and the corresponding mRNAs are targeted for degradation by the NMD pathway (for review, see He and Jacobson 2015). Using toe-printing analysis to map the positions of stable ribosomal complexes in yeast cell-free systems, it was noted that, in contrast to regular stop codons, ribosomes failed to be efficiently released when they encountered a PTC on the *CAN1* reporter mRNA (Amrani *et al.*^2004^). Subsequent addition of the elongation inhibitor cycloheximide allowed detection of additional signals that were attributed to 80S ribosomes with their P-sites centered on AUG codons in proximity to the PTC (Amrani *et al.*^2004^). This led to the suggestion that ribosomes that failed to be released from the PTC could migrate preferentially in the 5^΄^ direction (accounting for the designation retroREI), and reinitiate from nearby AUGs (Fig. 10). This event was shown to depend on PTC recognition by eRF1 and execution of peptide hydrolysis because the presence of defective eRF1 led to ribosomal stalling at the PTCs (Amrani *et al.*^2004^). This is consonant with the data from *in vitro* reconstituted termination on short uORF containing mRNAs where migration of 80S ribosomes in the 5^΄^ direction strictly depended on previous peptide release (Skabkin *et al.*^2013^). Even though migration to codons cognate to the deacylated P-site tRNA prevailed in this *in vitro* system, a small proportion of 80S ribosomes apparently lacking the deacylated tRNA could rebind Met-tRNA_i_^Met^ and migrate to upstream AUGs (Skabkin *et al.*^2013^)—which might also be the case in retroREI. Interestingly, inactivation of factors involved in NMD (Upf1 and Upf2) diminished the retroREI toe prints at upstream AUGs suggesting that, besides eRF1 recognition of the PTC as a stop codon, NMD factors—required to couple PTC recognition to mRNA decay—also contribute to efficient retroREI (Fig. 10). The physiological importance of retroREI is debatable, i.e. whether any benefit could be derived from synthesis of truncated proteins, often out of frame, from aberrant mRNAs that are being targeted for degradation to prevent the synthesis of potentially harmful truncated proteins. Nevertheless, the findings that ribosome recycling is inhibited at PTCs and that NMD factors are required for a scanning-like retromigration of post-termination complexes deserve additional investigation.

### Stop-Carry On or StopGo translation

Stop-Carry On or StopGo translation represents a rather peculiar REI mechanism where translation elongation arrest leads to termination at a specific sense codon (‘Stop’), release of the nascent polypeptide, and subsequent REI (‘Go’) at the next in-frame codon, resulting in the synthesis of a separate downstream protein product (Atkins *et al.*^2007^; Brown and Ryan 2010). In other words, within the coding sequence the synthesis of one specific peptide bond is skipped. The mechanism was first described in the FMDV (Foot and Mouth Disease Virus) apthovirus and in the EMCV (EncephaloMyoCarditis Virus) cardiovirus of the *Picornaviridae* family as a strategy used in the biogenesis of viral proteins (Donnelly *et al.*2001a,b; Brown and Ryan 2010). Since then it was found in many other positive-strand RNA viruses known for the production of multiple proteins from a single long ORF, including other mammalian *Picornaviridae* subgroups (erboviruses, teschoviruses and parechoviruses) and insect iflaviviruses and *Tetraviridae* and *Dicistroviridae* families (Luke *et al.*^2008^). Apparently, such unconventional REI events provide an alternative to proteolytic processing of a polyprotein in single-stranded RNA viruses. StopGo translation has also been identified in mammalian (rotaviruses), insect (cypoviruses) and crustacean (*Totiviridae*) double-stranded RNA viruses, and in sequences derived from non-LTR retrotransposons of trypanosomes and the purple sea urchin (Donnelly *et al.*2001a,b; Heras *et al.*^2006^; Luke *et al.*^2008^). Interestingly, in the latter species, this mechanism may also control cellular protein synthesis and was suggested to participate in the innate immune system (Brown and Ryan 2010).

**Figure 11.**
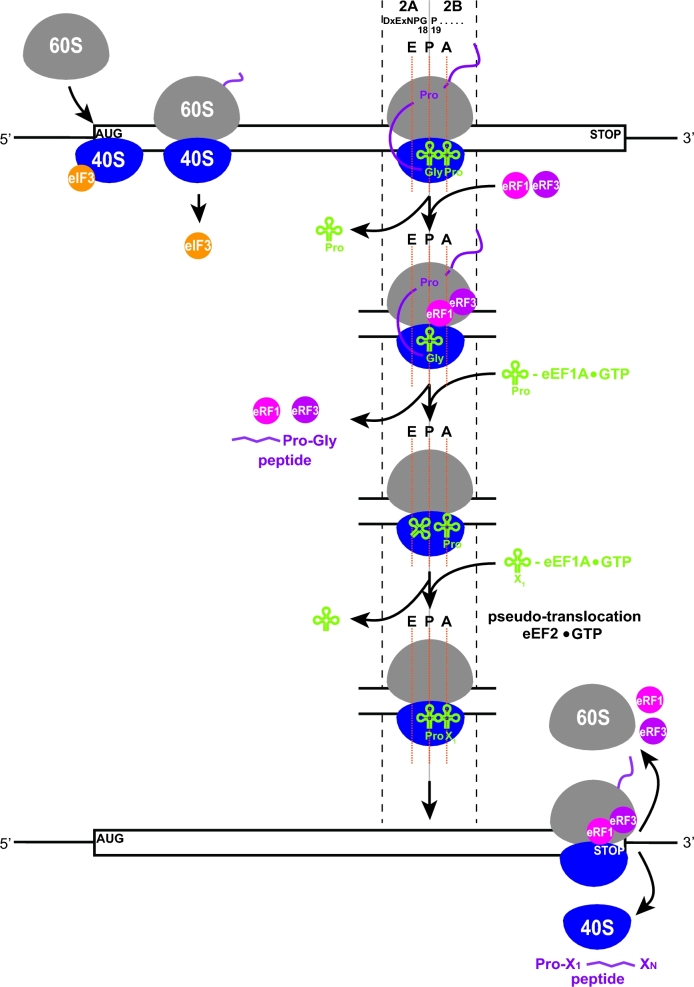
Model for the StopGo translation reinitiation. For details, see the main text.

In cardio- and apthoviruses, the StopGo occurs between segments 2A and 2B of the polyprotein that form the boundary between viral upstream capsid proteins and downstream RNA replication factors (Fig. 11). Alignment of their 2A sequences (which are only 18 aa long in apthoviruses compared to ∼150 aa in cardioviruses) led to identification of a conserved motif DxExNPG/P (where x is any amino acid) situated at the C-terminal end of 2A with the last conserved proline residue representing the N-terminal residue of the 2B segment (Fig. 11) (Donnelly *et al.*^1997^). Similar motifs were later found in other viral genera or cellular genes utilizing StopGo (Luke *et al.*^2008^; Brown and Ryan 2010). The 2A penultimate proline (proline-17; according to apthoviruses) and ultimate glycine (glycine-18) residues are completely conserved and essential among all active 2A and 2A-like sequences, while the 2B proline (proline-19) can be substituted by glycine at the expense of lower efficiency (Donnelly *et al.*2001a,b). Remarkably, 2A peptides work in all eukaryotic systems but not in prokaryotes (Ryan and Drew 1994; Donnelly *et al.*^1997^). Interestingly, phylogenetic analyses of 2A and 2A-like sequences indicated multiple, independent acquisitions of these sequences at different stages during virus evolution (Luke *et al.*^2008^).

In contrast to proteins analyzed from the FMDV-infected cells, where no full-length protein products including both 2A and 2B segments were observed, expression of artificial proteins from a bicistronic reporter construct with the 2A segment inserted in-frame between the two cistrons revealed an imbalance in the accumulated translation products (Donnelly *et al.*^1997^, 2001a,b). These and other experiments indicated that besides the 2A segment, its surrounding sequences also contribute to the efficiency of StopGo. In accordance with this, an upstream extension of the FMDV 2A segment to ∼30 aa by the authentic viral sequence regained 100% of the StopGo efficiency, no fusion products were observed and reporter proteins were produced in equimolar ratios (Donnelly *et al.*^1997^, 2001a,b).

The molecular mechanism of StopGo REI is distinctively associated with the specific nature of the essential glycine-18 and proline-19 residues. Toe-printing analysis and puromycin treatment indicated that the StopGo event might begin with ribosomal pausing at the end of the 2A-coding sequence, with the glycine-18 codon in the P-site and the proline-19 codon in the A-site (Fig. 11) (Donnelly *et al.*2001a,b; Doronina *et al.*^2008^). Ribosomal pausing and subsequent inhibition of the peptide bond formation is presumably caused by specific biochemical properties of these two residues, such as poor chemical attractiveness between their reaction centers or a spatially constrained conformation of proline-19. It was also suggested that the conserved proline-17 may help to reorient the glycyl-peptidyl-tRNA to disfavor peptide bond formation by adopting a tight-turn (Donnelly *et al.*2001a,b). Further constraints to peptide bond formation may be induced by sequences upstream of the 2A motif that could, depending on their length, establish interactions inside of the ribosomal exit tunnel and thus provoke ribosomal stalling (Donnelly *et al.*2001a,b).

More insights into StopGo REI were gained by indirect evidence suggesting that eRF1 and eRF3 contribute to this unusual termination event on sense proline codon (Fig. 11). In particular, depletion of eRF1 both *in vivo* and *in vitro* was accompanied by ∼30%–40% reduction in the efficiency of StopGo REI, increasing the amount of the full-length polyprotein (Doronina *et al.*^2008^). This implies that, under these specific circumstances, eRF1 recognizes sense proline codon as a stop codon and that its hydrolytic activity contributes to scission of the ester bond of the P-site glycyl-peptidyl-tRNA. In contrast, impairing the eRF3 GTPase activity prevented the majority of ribosomes from translating beyond 2A, substantially reducing the amounts of the downstream translation product (Doronina *et al.*^2008^). This indicates that while compromising eRF3 activity increases the efficiency of the sense-codon termination, it markedly impairs the ‘Go’ phase. Therefore, it was suggested that in order to circumvent the termination codon decoding function of eRF1 for the ‘Stop’ phase, hydrolysis of the nascent chain is uncoupled from GTP hydrolysis on eRF3 (GTP hydrolysis on eRF3 normally senses a perfect match between the stop codon and eRF1). In other words, hydrolysis of the nascent chain does not follow GTP hydrolysis on eRF3 as it does during canonical termination, but would occur independently of it. The delay in GTP hydrolysis on eRF3 may extend the occupancy of both eRFs on the ribosome, thereby increasing the time window for both ester bond hydrolysis and release of the 2A peptide from the ribosome (Doronina *et al.*^2008^; Brown and Ryan 2010). Whether the experimental set-up used in this study truly recapitulates what happens in the reality, however, remains to be seen.

In the current model of StopGo REI (Fig. 11), ribosomes translate the 2A sequence until glycine-18 has been incorporated into the nascent chain and its tRNA translocated into the P-site. This pauses the ribosome and enables the entry of eRFs into the A-site in place of the prolyl-tRNA. Subsequent hydrolysis of the glycyl-peptidyl-tRNA ester bond, uncoupled from GTP hydrolysis on eRF3, allows the release of the 2A peptide and dissociation of both eRFs from the halted ribosome followed by entry of the prolyl-tRNA to the A-site. Since there is only deacylated tRNA^Gly^ remaining in the P/E-site, pseudo-translocation promoted by eEF2 (Thompson 2012) is probably needed to transfer prolyl-tRNA to the P-site without peptide bond formation to begin the ‘Go’ phase. In support of this, early studies with the EMCV cardiovirus revealed that low levels of eEF2 prevented translation of products corresponding to proteins downstream of 2A, which could be overcome by the addition of purified eEF2 (Svitkin and Agol 1983). More work is needed to define the full spectrum of factors promoting/inhibiting StopGo REI and to understand its molecular mechanics—for example, how this mechanism prevents ribosomal recycling that normally follows termination by eRFs –including structural analysis of the StopGo complexes. It is noteworthy that the autonomous function and high efficiency of the StopGo REI mechanism in various eukaryotic translation systems have been extensively exploited for many practical purposes: in biotechnology to achieve stoichiometric co-expression of different proteins, in a recent remake of the dual luciferase reporter system detecting stop codon readthrough to avoid artifacts that can arise from translation of fused dual reporters (Loughran *et al.*^2017^), or for generation of vectors expressing multiple proteins from a single transcript for gene therapy and biomedical research (Luke *et al.*^2010^).

## CONCLUDING REMARKS AND FUTURE PROSPECTS

The cumulative evidence described in this review clearly demonstrates that translation-REI has many forms and takes place at practically any position within mRNAs. From the mechanistic point of view, the major difference is between REI promoted by 40S versus 80S post-termination complexes. To facilitate our attempt to describe all existing REI mechanisms, we have presented simplified models for each of them that emphasize the features that are at least partially shared by most representatives of each group.

For 40S-mediated REI, it is essential that, following translation termination, only the first step of ribosome recycling occurs, i.e. 60S dissociation catalyzed by ABCE1, to produce a post-termination 40S subunit bound to the mRNA at the stop codon of the uORF. This means that the second recycling step must be prevented to preserve the 40S-mRNA post-TC. At the same time, the ultimate deacylated tRNA should be released from the P-site to allow its replacement by Met-tRNA_i_^Met^, either in the form of eIF2-TC or possibly alone when DENR-MCT-1 (or Ligatin) is involved. Rebinding of eIF2-TC or Met-tRNA_i_^Met^ to the P-site is needed for recognition of the downstream AUG codon selected for REI (see below). There is now experimental evidence that eIF3 is a key factor capable of stabilizing interaction of the post-termination 40S subunit with mRNA, and might compensate for loss of the stabilizing codon:anticodon interaction involving the ultimate deacylated tRNA. In order to exert this function, eIF3 must be retained by the elongating ribosome during translation of ORF1, which is possible because its primary interactions with the 40S are confined to the solvent-exposed surface that remains accessible in the 80S ribosome (Fig. 2B and Fig. S2, Supporting Information). However, owing to stochastic dissociation of eIF3 from elongating 80S complexes, it can be retained at appreciable levels only during translation of short uORFs, which helps to explain the fact that the appreciable levels of REI occur without specialized mRNA-ribosome interactions only for short uORFs. Although eIF3 acts during primary initiation to stabilize PIC interactions with the mRNA, especially at the exit channel, its ability to markedly enhance REI following short uORF translation requires specialized mRNA sequences which it interacts with functionally (and perhaps physically) within the 40S post-TC, exemplified by particular REI-promoting sequences upstream of *GCN4* uORFs 1 and 2 (Fig. 2A).

In the case of REI following long ORFs, specialized mechanisms are required either to prevent the release of eIF3 from elongating ribosomes during the prolonged period required to translate the first long ORF or to bolster or substitute for the stabilizing role of eIF3 with specialized mRNA sequences that physically tether the 40S subunit to the mRNA. TAV-stimulated REI exemplifies the first case, wherein proteins without general functions in translation (TAV and RISP) collaborate to retain eIF3 on one or both subunits of the elongating 80S ribosome during translation of the first ORF, and so does the phosphorylation of eIF3h observed in *A. thaliana*. The TURBS-18S rRNA interaction exemplifies the second strategy. In contrast to the first strategy, however, the tethering of the 40S subunit to the TURBS element located at the 3^΄^ end of the first ORF imposes limitations on where REI can occur, restricting it to a start codon for ORF2 located fairly close to the ORF1 stop codon. Actually, these physical constrains imposed by the TURBS-18S rRNA interaction may explain an increased tolerance of the trapped ribosome for REI on non-canonical restart sites.

Because 40S REI generally utilizes an AUG start codon in the downstream ORF, Met-tRNA_i_^Met^ must be reacquired by the post-TC 40S subunit to allow recognition of the downstream AUG start codon by standard base pairing with the anticodon of Met-tRNA_i_^Met^. In the case of REI following short uORFs, this generally involves rebinding of the eIF2-GTP-Met-tRNA_i_^Met^ (eIF2-TC) as the 40S subunit traverses the intercistronic spacer between the uORF and main ORF. Upon rebinding of the eIF2-TC, traversing changes into genuine scanning for an AUG. In the case of highly structured intercistronic spacers, traversing/scanning of the 40S might require the activity of helicases to dissolve the mRNA secondary structures, which can be achieved by the eIF4F complex or perhaps other RNA helicases associated with eIF4F or the PIC. In TURBS-mediated REI, the established TURBS-40S interaction upon termination not only ensures that REI occurs close to the stop codon but also allows sufficient time for eIF2-TC reacquisition. In some instances, DENR-MCT-1 (or Ligatin) might promote the reacquisition of Met-tRNA_i_^Met^ instead of eIF2, either coupled with or uncoupled from ejection of the ultimate deacylated tRNA from the P-site, which remains to be experimentally tested. Other important questions that need to be addressed are when eIFs 1, 1A and 5 join the 40S ribosome *en route* to the next ORF and whether DENR-MCT-1 (or Ligatin) is capable of persisting on reinitiating ribosomes and substituting for the activities of the latter eIFs during scanning and AUG recognition at the downstream ORF.

In contrast to 40S-mediated REI, where several *cis-* and *trans*-acting factors functionally cooperate to prevent dissociation of mRNA from the 40S post-TCs, 80S-mediated REI can be viewed as a passive process stemming from either an elongation failure (in particular, an inability to form a peptide bond) or a termination/recycling failure (e.g. an inability to recycle the 80S post-TC upon termination on either regular or premature stop codons). These ‘failures’ may become advantageous under specific circumstances and are often exploited by numerous viruses. For 80S-mediated REI, the mRNA is stably anchored in the mRNA binding channel, locked in by the deacylated P-site tRNA base paired with the penultimate codon in the P-site; hence, there is no requirement for a dedicated mRNA retention mechanism. Upon polypeptide release and departure of both eRFs, the post-termination 80S ribosome may begin to traverse in either direction (even though upstream movement might be limited by an elongating 80S ribosome approaching the same stop codon from the 5^΄^ direction) and will eventually reinitiate when a ‘preferred’ codon appears in the A-site, the nature of which is probably determined by the identity of the P-site tRNA and the aa-tRNA-eEF1A-GTP ternary complex ternary complex corresponding to the A-site codon, as well as the conformational states of the post-termination 80S ribosomes. Considering that a substantial fraction of these unorthodox REI events are probably prevented from occurring by cellular surveillance factors such as Dom34/PELO, a key question that remains to be answered is whether the 80S-mediated REI events serve an important physiological role as opposed to being mere byproducts of translational malfunctioning.

Despite the tremendous progress that has been made recently in identification of *cis-*acting mRNA sequences and *trans-*acting factors that promote REI in various organisms, the molecular basis of their respective functions is only partly understood. Besides the classical ‘wet-lab’ approaches of genetics and biochemistry using *in vitro* reconstituted systems, structural studies revealing ‘snap-shots’ of complexes involved in particular REI mechanisms should substantially increase our understanding of how they rewire the translational cycle. Although there are many features that are shared among different REI mechanisms, the observed peculiarities may allow us to develop specific strategies for modulating their efficiencies, which—in the long run—could help cope with dysregulation of REI-mediated translational control and uORF polymorphism implicated in a variety of human diseases (Calvo, Pagliarini and Mootha 2009; Barbosa, Peixeiro and Romao 2013; Janich *et al.*^2015^).

## Supplementary Material

Supplementary Figures
