# Supplementary material for: Please do not recycle! Translation reinitiation in microbes and higher eukaryotes
Source: FEMS Microbiol Rev. 2017 Dec 21;42(2):165–92. doi: 10.1093/femsre/fux059 (PMC5972666; doi:10.1093/femsre/fux059)
Supplement: Supplementary Figures [file fux059_supp.pdf]

Please do not recycle!

Translation reinitiation in microbes and higher eukaryotes.

Stanislava Gunišová[1*], Vladislava Hronová[1], Mahabub Pasha Mohammad[1], Alan G. Hinnebusch[2*], and Leoš Shivaya Valášek[1*]

[1] Laboratory of Regulation of Gene Expression, Institute of Microbiology ASCR, Videnska 1083, Prague, 142 20, the Czech Republic

[2] Laboratory of Gene Regulation and Development, Eunice Kennedy Shriver National Institute of Child Health and Human Development, NIH, Bethesda, MD 20892, USA.

SUPPLEMENTARY DATA

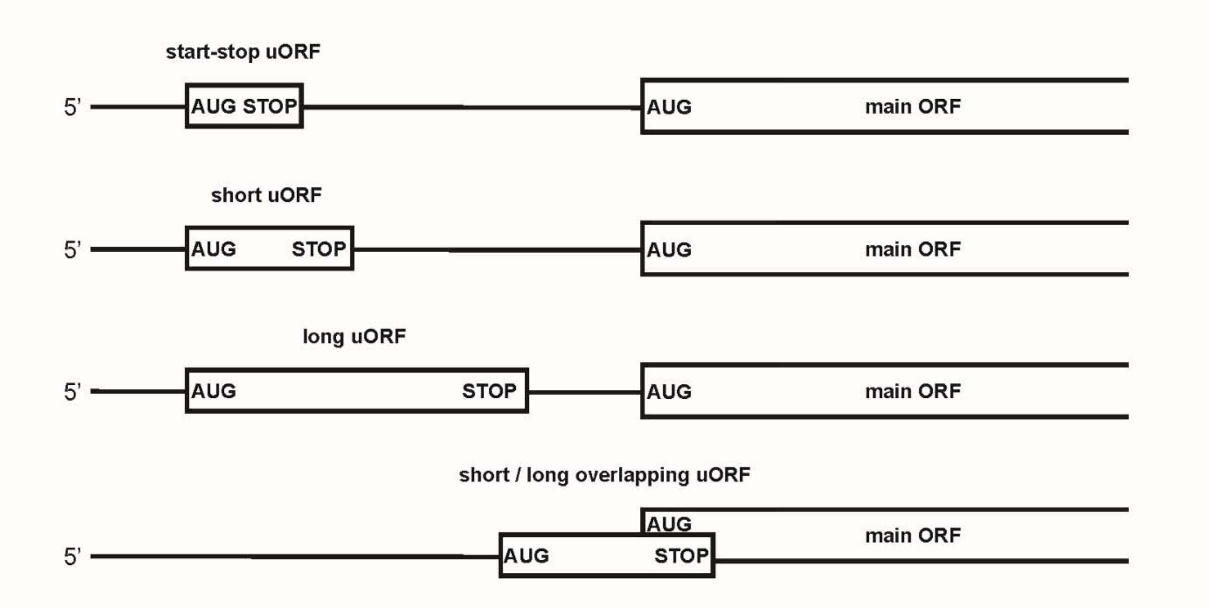

Supplementary Figure 1. Schematic illustrating various forms of upstream ORFs. Please see the main text for further details.

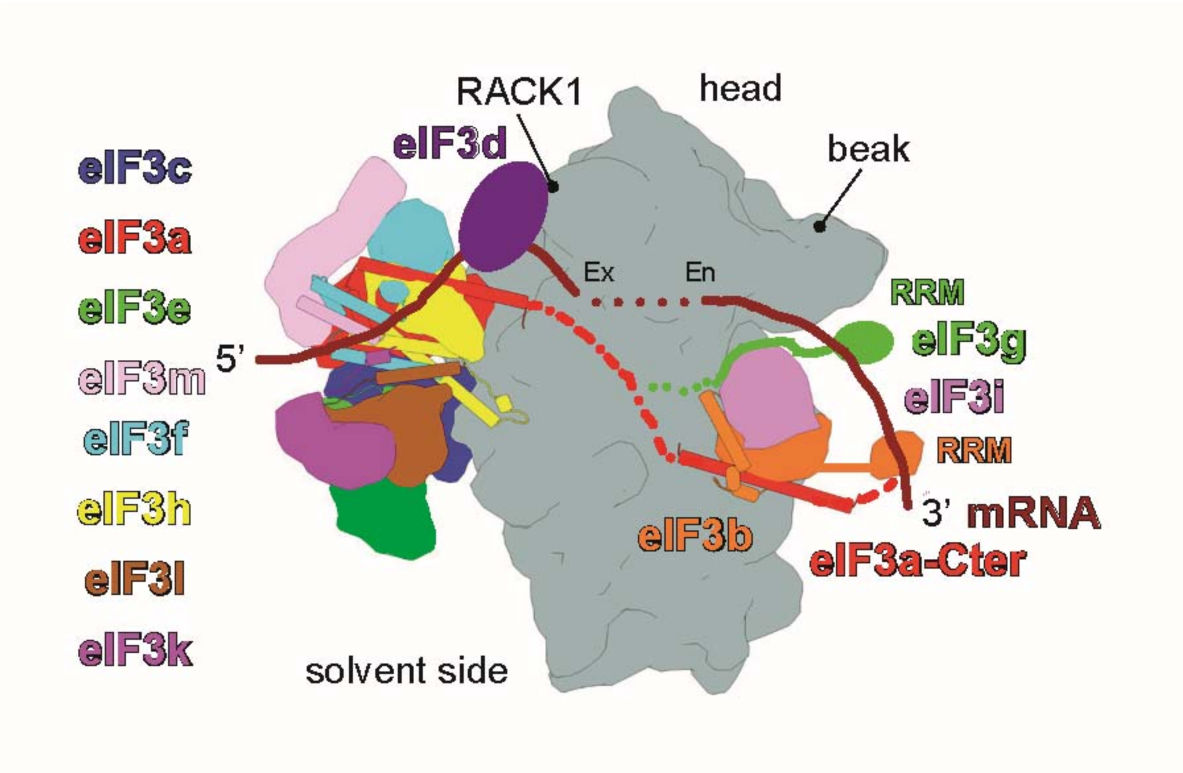

Supplementary Figure 2. Schematic representation of the arrangement of mammalian eIF3 subunits on the solvent-exposed side of 40S subunit (adapted from (Valasek *et al.*, 2017)). eIF3 binds to the solvent-exposed side with the octamer occupying the platform of the small ribosomal subunit connected with the eIF3b–g–i module (YLC) – sitting near the mRNA entry channel – *via* the extended C-terminal linker domain of eIF3a (a dashed red line indicates a predicted location of the eIF3a-CTD (eIF3a-Cter); placement of the eIF3g-RRM is also only our best guess). Figure includes only those domains of eIF3 subunits for which the structures are known. The 40S subunit is depicted in grey surface; all other subunits are labelled and colored variably. The eIF3 helical bundles fortifying the intersubunit interactions are represented as cylinders. The predicted path of mRNA is shown in dark red; Ex and En – mRNA exit and entry channels, respectively. For details please see the main text.

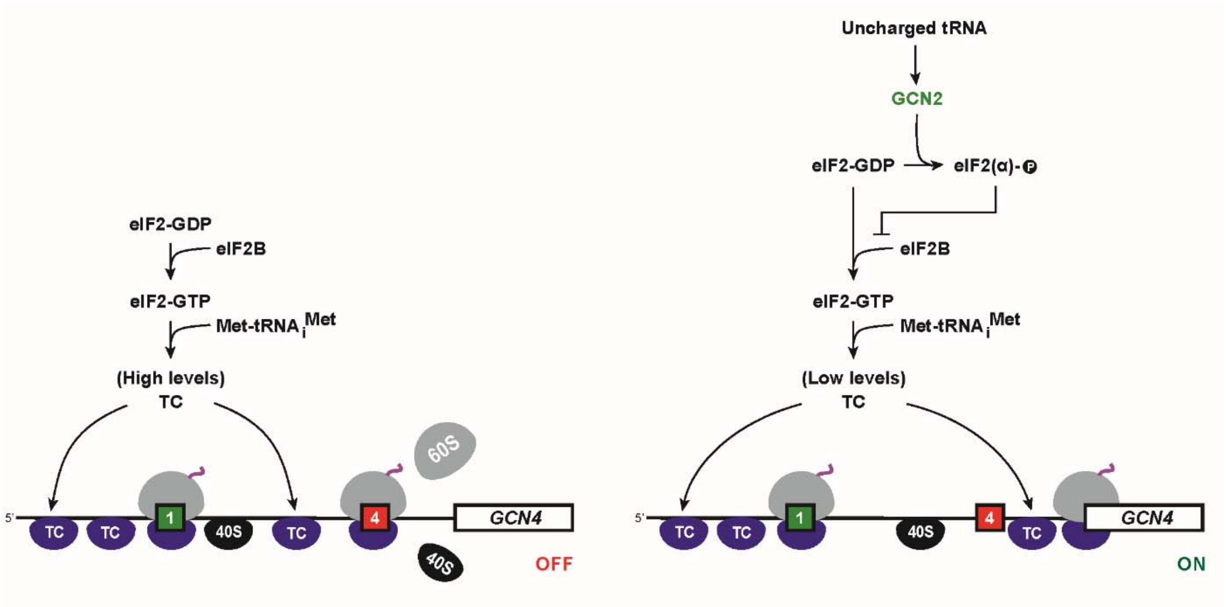

Supplementary Figure 3. Simplified model for the delayed REI mechanism on the *GCN4* mRNA under non-starvation *versus* starvation conditions depicting only uORFs 1 and 4; for details please see the main text (based on (Hinnebusch, 2005)).
